# Developmentally non-redundant SET domain proteins SUVH2 and SUVH9 are required for transcriptional gene silencing in *Arabidopsis thaliana*

**DOI:** 10.1007/s11103-012-9934-x

**Published:** 2012-06-06

**Authors:** Markus Kuhlmann, Michael Florian Mette

**Affiliations:** Leibniz Institute of Plant Genetics and Crop Plant Research (IPK) Gatersleben, Corrensstrasse 3, 06466 Gatersleben, Germany

**Keywords:** SET domain protein, RNA-directed DNA methylation, AtSN1, SUVH2, SUVH9

## Abstract

In plants, RNA-directed DNA methylation (RdDM) and related transcriptional gene silencing (TGS) involve members of the suppressor of variegation 3-9-homologous (SUVH) group of putative histone methyltransferases. Utilizing a reverse genetic approach in *Arabidopsis thaliana*, we demonstrate that two closely related *SUVH* members, *SUVH2* and *SUVH9*, act partially non-redundant in RdDM. DNA methylation, transcript accumulation and association with histone modifications were analyzed at the endogenous RdDM target *AtSN1* (a SINE-like retroelement) in *suvh2* and *suvh9* single as well as *suvh2* *suvh9* double mutants. *SUVH2* was found to be required for full DNA methylation at *AtSN1* in early seed development and was also higher expressed in seeds than at later developmental stages. *SUVH9* had its impact on RdDM later during vegetative development of the plant and was also higher expressed during that stage than at earlier developmental stages. The strongest reduction of RdDM at *AtSN1* was found in *suvh2* *suvh9* double mutant plants. Histone 3-lysine 9-dimethylation (H3K9me2) associated with *AtSN1* was reduced only in the simultaneous absence of functional *SUVH2* and *SUVH9*. Thus, *SUVH2* and *SUVH9* functions in RdDM and TGS are overlapping in spite of some developmental specialization. Pol V specific transcripts were reduced in *suvh2* *suvh9* plants. This might indicate a role of these SUVH proteins in Pol V complex recruitment.

**Electronic supplementary material:**

The online version of this article (doi:10.1007/s11103-012-9934-x) contains supplementary material, which is available to authorized users.

## Introduction

SET domain containing proteins can be divided into four different classes typified by the corresponding proteins of *Drosophila* E(Z), TRX, ASH1 and SU(VAR)3-9 (Baumbusch et al. 2001; Thorstensen et al. 2011). In *A. thaliana*, 15 genes similar to the *SU(VAR)3*-*9* were identified, of which 10 belong to the group of suppressor of variegation [SU(VAR)3-9] homologous genes (SUVH) defined by the presence of an internal YDG/SRA-domain and C-terminal pre-SET and SET domains. Among those, SUVH2 (encoded by *At2g33290*) and SUVH9 (encoded by *At4g13460*), show considerable sequence similarity (Naumann et al. 2005) and form a subgroup within the SUVH-family that is characterized by the absence of the catalytically important post-SET domain (Rea et al. 2000). SUVH2 has been reported to participate in gene silencing via heterochromatization (Naumann et al. 2005; Ay et al. 2009) and both, SUVH2 and SUVH9, are involved in DRM2-mediated de novo DNA methylation of particular target regions in *A. thaliana* (Johnson et al. 2008). The SET domain is essential for histone methyltransferase activity (Rea et al. 2000). Nevertheless, in vitro histone methylation tests were successful so far only for a subset of SUVH proteins, possibly due to rather specific substrate requirements of some of the family members. SUVH4, SUVH5 and SUVH6 readily methylate bovine H3 (Jackson et al. 2004; Ebbs and Bender 2006). SUVH5 additionally showed some activity on bovine H2A (Ebbs and Bender 2006). SUVH2 has been reported to methylate H4 in one study using reconstituted recombinant nucleosomes (Naumann et al. 2005), but was found inactive in a second study using bovine histones or *A. thaliana* nucleosomes (Johnson et al. 2008).

The second characterized domain in the SUVH proteins is the SET and RING associated (SRA) domain (Johnson et al. 2007, also termed YDG domain according to Baumbusch et al. 2001). This domain was shown to be responsible for the binding of proteins to methylated DNA in vitro (Rajakumara et al. 2011). The SRA domains of histone methyltransferases SUVH4 and SUVH6, which are linked to MET1- and CMT3-mediated maintenance of DNA methylation, have preferential affinity to methylated cytosines in CHG and CHH (H indicating A,C or T) context (Johnson et al. 2007). SUVH2 and SUVH9 are involved in de novo RNA-directed DNA methylation (RdDM) (Naumann et al. 2005; Johnson et al. 2008). The SRA domain of SUVH2 shows high affinity to methylated cytosines in CG motifs, while the SRA domain SUVH9 has the strongest affinity to methylated cytosines in CHH context (Johnson et al. 2008). Based on these results, Johnson et al. (2008) postulated that SUVH2 and SUVH9 may act in RdDM rather via binding to methylated cytosines in particular sequence contexts, than by actually methylating histones.

*AtSN1* (*A. thaliana* *SINE* like element 1) is a putative transposable retroelement present in 71 copies in the *A. thaliana* Col-0 reference genome (Myouga et al. 2001). A single copy of *AtSN1* located on chromosome 3 that is flanked by genic sequences has served as a model for the genetic analysis of RdDM of genomic sequences of *A. thaliana* in many studies (Zilberman et al. 2003; Xie et al. 2004; He et al. 2009a, b; Gao et al. 2010). Its internal region between the long terminal repeats (LTR) contains in total 44 cytosine sites; 11 in symmetric (4 CG sites, 7 CHG sites) and 33 in asymmetric (CHH) context. ~40 % of these cytosines are methylated in wild type plants. In symmetric context, cytosine methylation of *AtSN1* and other plant sequences, is primarily mediated by the DNA methyltransferase MET1 (Finnegan et al. 1993; Lindroth et al. 2001; Bartee et al. 2001) while methylation in asymmetric context is mainly mediated by DRM2 (RdDM pathway; Cao and Jacobsen 2002). In addition to the de novo DNA methyltransferase DRM2, the DNA dependent RNA polymerases Pol VI and Pol V contribute to RdDM and transcriptional suppression of *AtSN1* (Wierzbicki et al. 2008) by synthesis of siRNA homologous to the methylated region (Pontier et al. 2005). Further factors involved in RdDM are RDR2, DRD1 and DDM1 (Wierzbicki et al. 2009). Most likely, the RdDM pathway involves a self amplifying feedback loop of siRNA dependent DNA methylation and DNA methylation dependent Pol IV-RDR2 mediated formation of siRNAs (Law and Jacobsen 2010).

In wild type plants with functional RdDM, no *AtSN1* sense transcripts are found (Wierzbicki et al. 2009; Haag et al. 2009), although the internal LTR-flanking region of *AtSN1* shares structural similarity with promoters of tRNA (Box A and Box B) and of genes typically transcribed by DNA-dependent RNA Pol III (Myouga et al. 2001). Formation of *AtSN1* sense transcripts is seen in mutants with disturbed RdDM such as *nrpe1, ago4* and *dms3*. As the transcriptional activation was not associated with acetylation of H3 at lysines 9 and 14 or Pol II association at *AtSN1*, it is assumed that *AtSN1* transcription is Pol III-dependent (Wierzbicki et al. 2009). *AtSN1* silencing in wild type plants is associated with complementary and adjacent antisense transcripts, which are most likely products of Pol V (Wierzbicki et al. 2008) which are missing in *nrpe1* mutant plants (NRPE1 is the largest subunit of the Pol V complex) and reduced in *rdm3* mutants (He et al. 2009a, b, c). Interaction of NRPE1 with the *AtSN1* region was shown by chromatin immunoprecipitation (ChIP) (Rowley et al. 2011).

In the silenced state, the *AtSN1* region is associated with high levels of repressive histone marks such as H3K9me2 and H3K27me1, while acetylation of histone 3 is mainly absent (Wierzbicki et al. 2008). Addionally, association of *AtSN1* with H3K4me2, a mark associated with active chromatin was described, defining this region as “intermediate heterochromatin” (Habu et al. 2006). In plants without functional Pol V, the level of H3K9me2 remained unaltered in the internal *AtSN1* region (Wierzbicki et al. 2008).

Here, we show that the two putative histone methyltransferases SUVH2 and SUVH9 are differentially expressed during plant development and contribute, at least in part, non-redundantly to RdDM. SUVH2 plays a major role at the seedling stage and SUVH9 during vegetative growth. Nevertheless, both are important for transcriptional silencing and persistence of repressive histone marks.

## Materials and methods

### Plant material and growth conditions

The *A. thaliana* accession Col-0 was used in all experiments. The *suvh2* T-DNA insertion lines were ordered from NASC (Nottingham Arabidopsis Stock Centre, T-DNA insertion line Gabi_kat_516A07, Suppl. Figure 2). The *suvh9* T-DNA insertion line was ordered from NASC (T-DNA insertion line SALK_048033). Double mutant *suvh2* *suvh9* line was generated by crossing Gabi_kat_516A07 and SALK_048033 and genotyping of the F3 generation. SUVH2 over-expression plants were obtained from the laboratory of G. Reuter (Martin-Luther University Halle-Wittenberg, Germany). These plants contain a strong constitutive expressed myc-tagged *SUVH2* gene controlled by the CaMV35 promoter. Plants with enhanced level of SUVH2 were selected by their dwarf phenotype, curled leaves and delayed senescence.

Plants were cultivated on soil at 21 °C under a 16 h light/8 h dark (long day) regime for propagation and seed production or under an 8 h light/16 h dark (short day) regime to generate material for RNA and DNA analysis. All seeds were stratified before germination for 3 days at 4 °C in the dark. Kanamycin and phosphinotricin (BASTA^®^) resistance of the plants was tested on germination medium (1/2 MS salts; 10 g/l sucrose) plates with 40 mg/l kanamycin or 20 mg/l Glufosinate-ammonium under long day conditions.

Primers used for genotyping: Supplementary Table.

### RNA analysis

Total RNA samples for transcript measurements were extracted from seedlings grown for 1 week on GM medium under long day conditions and from rosette leaves of plants grown for 6–8 weeks under short day conditions. For analysis of RNA in imbibed seeds, seeds were soaked in water over night at RT. RNA was prepared in triplicates from 100 mg of seedlings grown on agarplates. Total RNA and small RNA were extracted from 1 g tissue with the mirVana™ miRNA Isolation kit (Ambion) according to manufacturers protocol E. and F. II. cDNA was prepared using RevertAid™ H Minus M-MuLVRT (Fermentas) according to manufacturers protocol. mRNA and AtSN1-A analysis was performed on oligo dT transcribed cDNA, AtSN1-B (and C) on cDNA using specific primers. cDNAs were quantified by the RT-qPCR method using the iCycler (Bio-rad) and the iQ™ SyBR^®^ Green Supermix (Bio-rad). Program: 1: 5′ 95 °C; 2: 15″ 95 °C; 3: 30″ 65 °C; 4: 30″ 72 °C; 5: goto2 40×; 6: Melting curve 65 °C 10″ +0.5 °C 60 repeats; 7: 4 °C.

Primers used for qPCR are defined in the Supplementary Table.

**Fig. 1:**
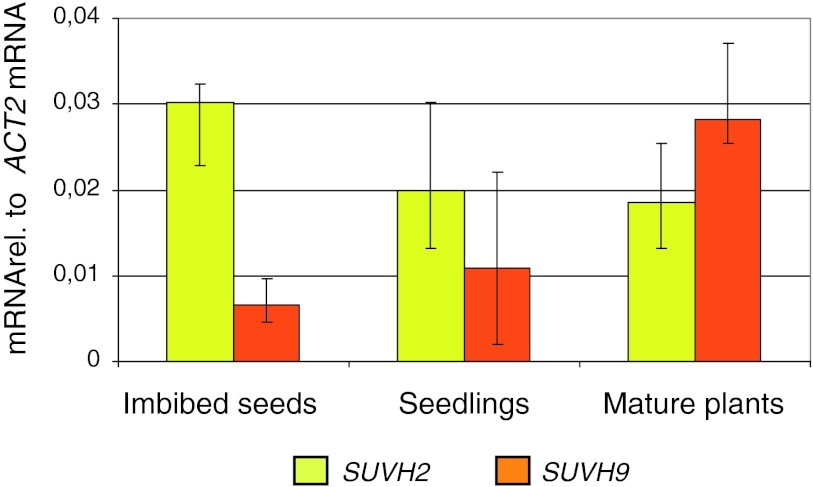
Differential expression of *SUVH2* and *SUVH9* in wild type *A. thaliana*. *SUVH2* (*yellow*) and *SUVH9* (*orange*) mRNAs were quantified relative to *ACT2* mRNA at 3 different developmental stages using RT-qPCR: Imbibed seeds: 24 h imbibing of the seeds in water, Seedlings: grown for 7 days on agar plates after stratification, and Leaves: leave tissue from plants grown for 6 weeks on soil. The depicted *bars* give the median and the *error bars* the total deviation of 5 independent biological samples

For each genotype, 5 individual plants were analyzed. Mid values are indicated as bar heights, standard deviation of values as error bars (Fig. 1, Suppl. Figure 3).

*ACTIN*2 (*ACT2*; *At3g18780*) and *PHOSPHOFRUCTOKINASE* (*PFK*; *At4g04040*; transcripts only present in seedlings and leaf tissue) were used as reference genes (Correlation Coefficient and PCR efficiency: Suppl. Figure 8).

### Northern blot analysis of siRNA/miRNA

Small RNA were detected by Northern blot analysis of a RNA fraction (10 μg per lane for miRNA 157a and 50 μg per lane for *AtSN1* siRNA) enriched for small molecules. Hybridization was performed as described by Mette et al. (2005) using a ^32^P-labeled *AtSN1* antisense transcript.

### DNA methylation analysis (Bisulfite sequencing)

Genomic DNA for methylation analysis was extracted with a DNeasy Plant Maxi Kit (Qiagen, Hilden, Germany) from rosette leaves of individually genotyped plants grown for 6–8 weeks under short day conditions and from seedlings grown on GM medium for 1 week under long day conditions.

Bisulfite-mediated chemical conversion of DNA was done using a Qiagen EpiTect Bisulfite Kit (Qiagen, Hilden, Germany) following the manufacturers instructions. Amplicons were transformed with the help of the StrataClone™ PCR Cloning Kit. Sequencing of single clonal colonies was done with the Cyclereader Auto Sequencing Kit (Fermentas, St.Leon-Rot, Germany) and Licor 4300 DNA Analyzer.

Statistical analysis was performed using Chi-square test comparing the number of methylation events out of all possible cytosine sites detected by bisulfite sequencing. Standard deviation is calculated by QUICKBASIC Program for Exact and Mid-P Confidence Intervals for a Binomial Proportion (see Suppl. Fig 4a–h).

Primers used for amplification of converted target regions are defined in the Supplementary Table.

### Chromatin immuno precipitation (ChIP)

The ChIP was performed according Jovtchev et al. (2011), Gendrel et al. (2005) and Lawrence and Pikaard (2004) with modifications. Four g of 1 week old seedlings were used for crosslinking by 1 % w/v formaldehyde solution under vacuum for 15 min (seedlings) or 10 min (leaves). Crosslinking was quenched with 2.5 ml 2 M glycine under vacuum for five min. The plant material was homogenized in liquid nitrogen and suspended in 30 ml extraction buffer. The extract was filtered through 2 layers of Miracloth and centrifuged at 1,800*g* for 20 min at 4 °C. The pellet was resuspended in 1 ml of extraction buffer 2 and centrifuged at 16,000*g* for 10 min. The pellet was resuspended in 300 μl extraction buffer 3 and overlayed on 300 μl of extraction buffer 3. The nuclei are collected by centrifugation at 16,000*g* for 1 h. The pellet was resuspended in 300 μl of cold nuclei lysis buffer. The sonication of the chromatin was performed using a Diagenode Disruptor for 6 cycles with 30 s of high energy sonication and 30 s break at 4 °C. Nuclear debris was removed by centrifugation at 16,000*g* for 10 min. One aliquot of the chromatin extract was used for the input and the others were diluted 1:10 in ChIP dilution buffer. The immunoprecipitation was performed with Dynabead Protein A (Dynal) coupled antibodies. H3: #39163 Histone H3 C-terminal rabbit pAB, Activemotif; H3K4me3: #39159 Histone H3 trimethyl Lys4 Rabbit pAB, Activemotif; H3K9me2: mouse monoclonal [mAbcam1220), Abcam; H3K27me3:; H4K20me1: ab9051, Rabbit polyclonal Histone H4 (monomethyl K20), Abcam were used.

After overnight incubation at 4 °C, the coupled beads were washed 2 times 5 min each with low salt buffer :150 mM NaCL, 0.1 % SDS, 1 % Triton X100, 2 mM EDTA, 20 mM Tris–HCl (pH 8.1); high salt buffer: 500 mM NaCL, 0.1 % SDS, 1 % Triton X-100, 2 mM EDTA, 20 mM Tris–HCl (pH 8.1); LiCl wash buffer: 0.25 LiCl, 1 % NP40, 1 % Na deoxycholate, 1 mM EDTA, 10 mM Tris–HCl (pH 8.1) and TE buffer: 10 mM Tris–HCl (pH 8.), 1 mM EDTA. The DNA was eluted twice by incubation with 250 μl elution buffer at 65 °C for 15 min.

Primers used for quantitative amplification of genomic regions after ChIP are defined in the supplementary table.

## Results

### *SUVH2* and *SUVH9* are differentially expressed during *A. thaliana* development

Transcripts of SET-domain protein family genes *SUVH2* (*At2g33290*) and *SUVH9* (*At4g13460*) are detectable throughout the lifecycle of *A. thaliana,* but show different expression levels when material of different stages of plant development is compared (Suppl. Figure 1). In seeds imbibed in water for 1 day, *SUVH2* expression was highest and decreased during seedling development and in mature leaves of 6 week old plants grown under short day conditions (Fig. 1). *SUVH9* showed an expression pattern inverse to that of *SUVH2*, i.e., a low *SUVH9* transcript abundance in seeds and a higher one in seedlings and leaves of mature plants. The observed changes in *SUVH2* and *SUVH9* expression could be due to differential gene regulation over developmental time or, provided that *SUVH2* and *SUVH9* are preferentially expressed in different cell types, might reflect the different cell type-composition of the analysed tissues.

### Single *suvh2* and *suvh9* mutations affect RdDM to different extent at different developmental stages

To test the significance of this differential expression of *SUVH2* and *SUVH9*, we applied a reverse genetic approach. T-DNA segregation analysis, sequencing of the PCR-amplified T-DNA integration locus (Suppl. Figure 2) and transcript quantification by RT-qPCR (Suppl. Figure 3) in T-DNA insertion lines identified the loss-of-function alleles *suvh2* (GABI-Kat 516A07; Rosso et al. 2003) and *suvh9* (SALK_048033; Alonso et al. 2003; Johnson et al. 2008) that both contained a single T-DNA insertion locus and showed robust specific reduction of *SUVH2* and *SUVH9* transcripts, respectively. A *suvh2* *suvh9* double mutant line was obtained by crossing of the single mutant lines followed by self-pollination of F_1_ and F_2_ plants. Homozygous *suvh2* *suvh9* plants were identified in the F_3_ generation using PCR-based markers selective for T-DNA insertion and wild type alleles. For all following analyses, material from homozygous F_4_ and F_5_ plants was used.

**Fig. 2:**
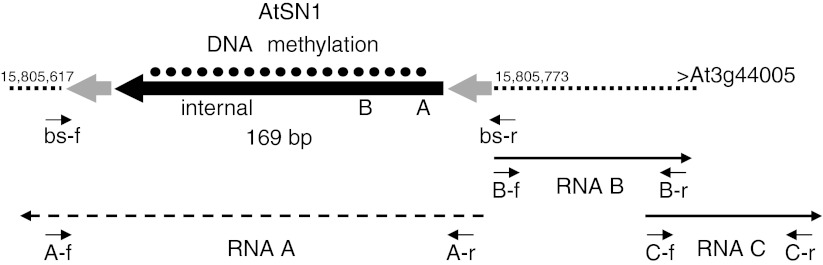
*AtSN1* as a model target of RNA-directed DNA methylation and transcriptional silencing. Schematic overview for the genomic region including *SINE*-like element *AtSN1* located on chromosome 3 next to *At3G44005*. LTRs (*bold grey arrows*) flank the inner region (*bold black arrow*) that contains two putative promoter-like structures, *Box A* and *Box B*, and the internal region with homology to *tRNA* genes. *Black dots* indicate the region in which methylated cytosines are detected in wild type. Positions of primers used for amplification after bisulfite conversion bs-f and bs-r indicate the region analyzed for DNA methylation. The *dotted arrow* shows the putative extent of silenced transcript A, the *black arrows* indicate Pol V derived transcripts B and C. Positions of primers used for RT-PCR are marked by *A-f*, *A-r*; *B-f*, *B-r*; *C-f*, *C-r*

**Fig. 3:**
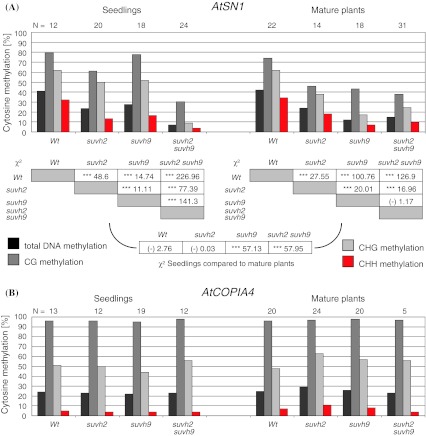
*AtSN1* and *AtCOPIA4* DNA methylation in *suvh2*, *suvh9* and *suvh2* *suvh9* mutants. DNA methylation was determined by bisulfite sequencing of genomic DNA from 7 day old seedlings or leaves of mature 6 week old plants. Individual clones (N) were sequenced for the different genotypes. The *bars* mark the levels of DNA methylation in percent of methylated cytosines relative to total cytosines, with black indicating all cytosines, *dark grey* CG context, *light grey* CHG context and *red* indicating CHH context. Wt indicates wild type plants. **a** DNA methylation in *AtSN1*. Significance of differences in total DNA methylation between the different genotypes were checked by Chi Square tests (table, ***p < 0.001 and ANOVA, data not shown). **b** DNA methylation at *AtCOPIA4*

The dispersed repetitive element *AtSN1* is known to undergo persistent RNA-directed DNA methylation and transcriptional silencing in wild type *A. thaliana* Col-0 throughout almost the whole life cycle of the plant. A particular copy of *AtSN1* located on chromosome 3 between annotated genes *At3g44000* and *At3g44005* (Fig. 2), nucleotides 15,805,617–15,805,773 of chromosome 3 (NCBI nucleotide sequence NC003074), has been used in several studies to check the effect of mutations on RdDM at endogenous sequences (Wierzbicki et al. 2008, 2009; Johnson et al. 2008). DNA methylation at this copy was determined by bisulfite sequencing of genomic DNA from seedlings and leaves of mature 6 week old wild type plants, *suvh2* and *suvh9* single mutant as well as *suvh2* *suvh9* double mutant lines (Fig. 3a, Supplementary Fig. 4A–H). Methylation levels were calculated in percent methylated cytosines of total cytosines, cytosines in CG, in CHG and in CHH context. In wild type plants, *AtSN1* DNA methylation was essentially the same in seedlings and leaves. Methylation was strongest reduced in *suvh2* *suvh9* double mutants. This reduction affected cytosines in all contexts and was rather similar in seedlings and leaves from mature plants. Thus, SUVH2 and SUVH9 have redundant roles, as the double mutant line displays a more pronounced effect on RdDM than the single mutants. Nevertheless, in seedlings, the *suvh2* mutation caused a stronger reduction of total DNA methylation than the *suvh9* mutation. This seemed mainly due to a stronger effect of *suvh2* on CHH context methylation. In contrast, a stronger reduction of total DNA methylation in the *suvh9* compared to the *suvh2* was found for leaves from mature plants. During development of seedling to mature plants, the level of DNA methylation in the *suvh9* mutant is consistently reduced, while it remains constant in the *suvh2*. Consistent with the peaks of *SUVH2* and *SUVH9* transcripts in seedlings and leaves of mature plants, respectively, SUVH2 seems to be more important for RdDM in seedlings and SUVH9 in mature leaves. Thus, SUVH2 and SUVH9 act at least in part developmentally non-redundantly over the plant life cycle.

*AtSN1* methylation was also determined in leaves from a line hemizygous for a *Pro35S*-*mycSUVH2* over-expression construct (Naumann et al. 2005; Ay et al. 2009). Despite their dwarf habitus typical for increased *SUVH2* transcript levels, *Pro35S*-*mycSUVH2* plants did not show an obvious increase in *AtSN1* methylation (Suppl. Figure 5). Thus, SUVH2 is required for RdDM of *AtSN1*, but is not the rate limiting factor if functional *SUVH2* and *SUVH9* alleles are present.

The GC-rich retroelement *AtCOPIA4* (At4g16870) is hypermethylated in the *A. thaliana* genome, mainly due to maintenance methylation involving MET1 and SUVH4 (Johnson et al. 2007). *AtCOPIA4* methylation is hardly affected in mutants of RdDM components. We performed bisulfite sequencing of an *AtCOPIA4* element containing 83 cytosines (14 in CG, 6 in CHG and 63 in CHH context) (Fig. 3b, Suppl. Figure 4 I to P) in order to test for effects *suvh2* and *suvh9* mutations on its DNA methylation. No obvious changes of DNA methylation were found for all genotypes in seedlings and leaves from mature plants indicating that *COPIA4* methylation is apparently not affected by mutants of the RdDM pathway.

### Only the *suvh2suvh9* double mutant shows released silencing of *AtSN1* sense transcription

**Fig. 4:**
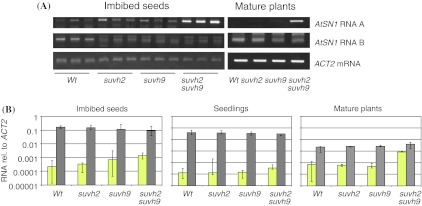
*AtSN1*-derived transcripts in *suvh2*, *suvh9* and *suvh2* *suvh9* mutants. *AtSN1* transcripts were determined relative to *ACT2* and *PFK* mRNA in total RNA from imbibed seeds, 7 day old seedlings or leaves of mature 6 week old plants by RT PCR-based methods with primers specific for transcripts A and B. **a** Semiquantitative PCR analysis of *AtSN1* transcripts A and B in imbibed seeds and leaves in comparison to *ACT2* mRNA. **b** RT-qPCR analysis of *AtSN1* transcript A (*yellow*) and *PFK* mRNA (*grey*) in imbibed seeds, leaves and seedlings relative to *ACT2* mRNA. The *bars* indicate the median of 5 biological samples measured in technical duplicate; the *error bars* indicate the total deviation among samples

The *AtSN1* region is the origin of three different transcripts (Fig. 2). In wild type plants, the *AtSN1* region is silenced and Pol III dependent transcription of sense transcript (A) is suppressed, whereas Pol V dependent antisense transcripts (B and C) are detectable (Wierzbicki et al. 2008). Thus, sense transcript A and antisense transcripts B and C were supposed to be mutually exclusive (Wierzbicki et al. 2008). We determined *AtSN1* transcripts A and B by reverse transcription combined with *ACT2* mRNA in RNA extracted from imbibed seeds and leaves of 6 week old wild type plants, *suvh2,* *suvh9* and *suvh2* *suvh9* double mutant lines (Fig. 4a). Consistent with Wierzbicki et al. 2008, the level of transcript A was low and that of transcript B was high in wild type seeds and leaves. The inverse pattern was found for the *suvh2* *suvh9* double mutant, where transcript A was increased and transcript B was reduced. The results for *suvh2* and *suvh9* single mutants were less clear. The increase of transcript A was not prominent, and transcript B was somewhat reduced in imbibed seeds. The effects of mutations in *suvh2* and *suvh9* on *AtSN1* transcript A levels were confirmed by reverse transcription combined with quantitative PCR (RT-qPCR; Fig. 4b). In comparison to wild type, *AtSN1* transcript A levels all in *suvh2* *suvh9* double mutants were increased 14 fold in imbibed seeds, 6 fold in seedlings and threefold in leaves of mature plants. Thus, with regard to their effects on *AtSN1* transcripts, SUVH2 and SUVH9 seem to act redundantly. The release of silencing of *AtSN1* in *suvh2* *suvh9* double mutants was associated with a decrease of siRNAs that targets *AtSN1* (Suppl. Figure 6).

To investigate the involvement of SUVH2 in silencing of Pol II dependent transcription, we used of a well characterized two component transgene system mediating RNA-directed transcriptional gene silencing (TGS; Aufsatz et al. 2002; Fischer et al. 2008). Although impaired reporter gene silencing was seen in the *suvh2* mutant (Suppl. Figure 7), this observation turned out to be inconclusive due to the structure of the used *suvh2* T-DNA insertion allele. Line GABI-Kat 516A07 contains two T-DNA copies in a tail-to-tail arrangement (Suppl. Figure 2). The resulting inverted repeat structure of *CaMV* *Pro35S* sequences leads to TGS of the silencer *Pro35S*-*NOSpro*-*proNOS* construct. Such cross-silencing effects were already reported for other T-DNA insertion lines (Daxinger et al. 2008).

### Release of *AtSN1* sense transcription in *suvh2suvh9* is correlated with altered histone modification

**Fig. 5:**
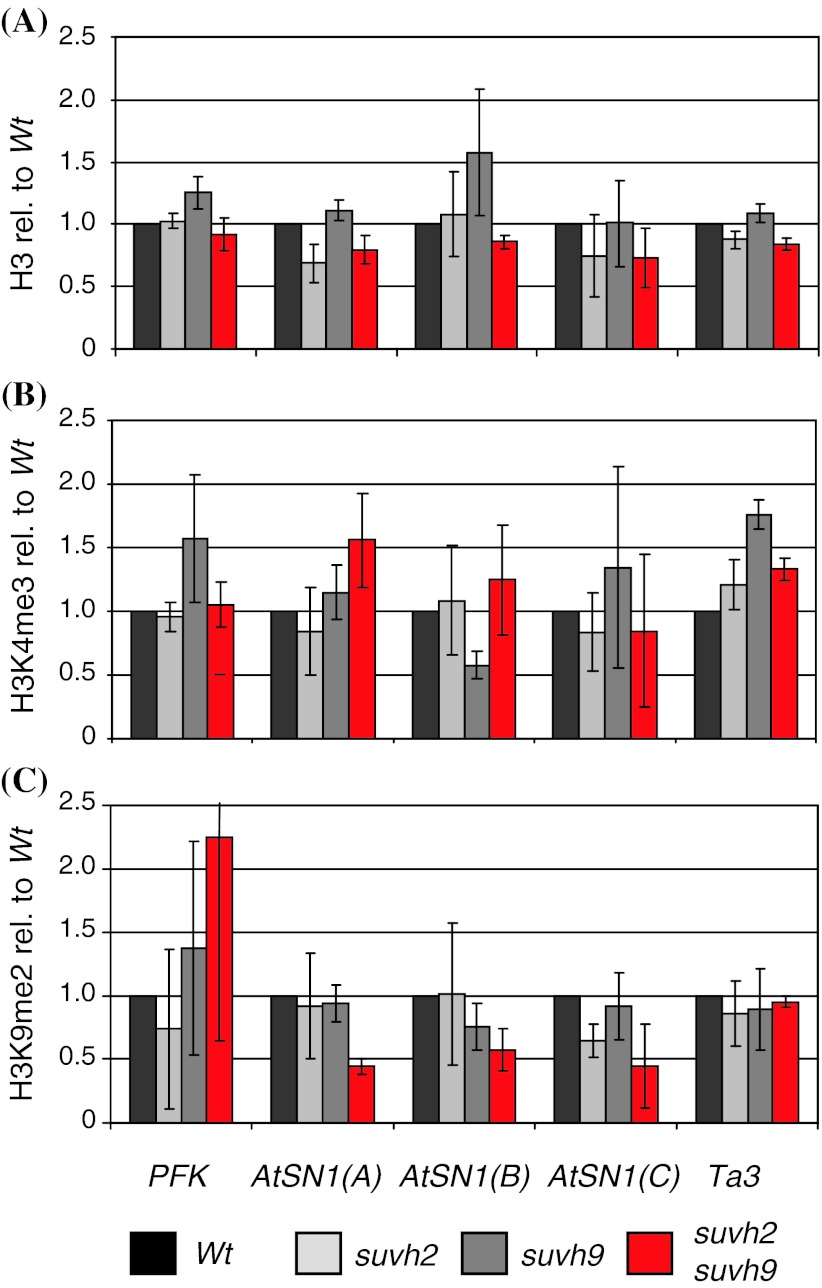
*AtSN1*-associated histone modifications in *suvh2*, *suvh9* and *suvh2* *suvh9* mutants. Chromatin immunoprecipitation combined with quantitative PCR (ChIP-qPCR) was performed on 1 week old seedling with antibodies specific to H3, H3K4me3 and H3K9me2 and primers specific for *PFK*, *AtSN1* regions A, B, C and *Ta3*. Wild type values were set to 1.0 and values for mutants were calibrated accordingly.* Black* indicates wild type,* light-grey* *suvh2* single mutants, *dark-grey* *suvh9* single mutants and red *suvh2 suvh9* double mutants. Quantification by qPCR was performed in duplicates. *Bars* indicate the mean; *error bars* the standard deviation from 3 independent experiments. **a** H3, **b** H3K4me3, **c** H3K9me2

DNA hypermethylation and transcriptional silencing are usually associated with increased nucleosome density and histone modifications typical for repressed chromatin such as H3K9me2 (Pecinka et al. 2010). Thus, chromatin immunoprecipitation combined with quantitative PCR (ChIP qPCR) was performed to quantify H3K4me3 and H3K9me2 associated with different regions of *AtSN1* in 7 day old seedlings (Fig. 5). To determine nucleosome density, we used an antibody specific for H3 irrespective of modifications. Primers were designed to separately analyze *AtSN1* regions corresponding to sense transcript A and to antisense transcripts B and C (Fig. 2). Wild type values were set to 1.0, and values for mutants were calibrated accordingly. As controls, the highly transcribed gene *PFK* typical for active and the retroelement *Ta3* typical for inactive chromatin were used (Johnson et al. 2002; Mathieu et al. 2003, 2005). As *PFK* shows very little association with H3K9me2, ChIP-qPCR for this mark at *PFK* is in particular error-prone. After calculation of relative values for mutants in comparison to wild type, this high initial errors result in a high deviation of values. The analysis of H3 density at *AtSN1*, *PFK* and *Ta3* did not show consistent differences between wild type and mutant plants that would indicate altered nucleosome density at these sequences in *suvh2* or *suvh9* mutants. If at all, a tendency for lower nucleosome density at *AtSN1* might be seen in the *suvh2* *suvh9* double mutant, which would be concordant with the increased transcriptional activity. H3K4me3 as a histone mark associated with actively transcribed chromatin (Fuchs et al. 2006; Moghaddam et al. 2011) was slightly increased in the *suvh2* *suvh9* double mutant at the *AtSN1* region homologous to transcript A. No consistent change was seen, neither for other sequences, nor in single *suvh2* and *suvh9* mutants. H3K9me2 is typical for transcriptional inactive chromatin (Stancheva 2005). In the *suvh2* *suvh9* double mutant, it was found reduced in the regions A, B and C of *AtSN1*. The reduction of H3K9me2 was more evident than the reduction in nucleosome density, indicating that the measurements reflected a genuine reduction of H3K9me2 rather than a reduction of nucleosome density. Again, no consistent changes were seen for other sequences analyzed, and also not in single *suvh2* and *suvh9* mutants. Thus, with regard to their effects on H3K4me3 and H3K9me2 density at transcribed *AtSN1* regions, SUVH2 and SUVH9 seem to act mainly redundantly. Thus, SUVH2 and SUVH9 non-redundancy seems limited to their role in DNA methylation. H3K27me2 and H4K20me1 were also analyzed at *AtSN1*, but no obvious changes were found between mutants and the wild type (data not shown).

## Discussion

During plant reproduction, processes of genome-wide epigenetic reprogramming are taking place (Slotkin et al. 2009). This can include changes of histone modifications, DNA methylation and siRNA levels. Nevertheless, potentially harmful elements such as *AtSN1* stay transcriptional silenced throughout development. Both SET domain proteins SUVH2 and SUVH9 are required for complete transcriptional silencing of AtSN1, acting in a seemingly redundant way. However, with regard to RNA-directed DNA methylation, SUVH2 and SUVH9 activities are at least in part developmentally non-redundant. This might be related to the different binding properties of the two proteins for methylated DNA (Johnson et al. 2008). Taking into account the reported in vitro binding preferences of the SUVH2 (methylated cytosines in CG context) and SUVH9 (methylated cytosines in CHH context) and the ratio of potentially methylated cytosines in these contexts in *AtSN1* (4 sites in CG vs. 33 CHH context), one would predict that loss of SUVH9 should have a stronger impact than loss of SUVH2, irrespective of the developmental stage of sampled plants. The effects of the single *suvh2* and *suvh9* mutations on AtSN1 methylation levels observed in our study do not meet this expectation. Rather, from the observed reduction of DNA methylation in *suvh2* seedlings and *suvh9* mature leaves, we would conclude that the differential expression patterns of *SUVH2* and *SUVH9* can explain their non-redundant involvement in RdDM over development. The high expression of the *SUVH2* in imbibed seeds indicates a possible role in the epigenetic reprogramming during early development. Stage-specific regulation of *SUVH2* might explain the delayed senescence phenotype observed by Ay et al. (2009) due to ectopic SUVH2 expression during late development and the resulting mis-regulation of senescence controlling genes.

In *A. thaliana* interphase nuclei, most heterochromatin resides in structures called chromocenters, which are characterized by highly methylated DNA and specific marks such as histone H3K9me2 on the molecular level (Soppe et al. 2002). A clear reduction of histone H3K9me2 at chromocenters has been reported for example for a *suvh4* *suvh5* *suvh6* triple mutant (Johnson et al. 2008; Jovtchev et al. 2011). For a *suvh2* single mutant a reduction of H3K9me2 at chromocenters of young leaves has been reported (Naumann et al. 2005), while for the same allele in a *suvh2* *suvh9* double mutant no immunologically detectable change of H3K9me2 in chromocenters was reported in another study (Johnson et al. 2008). This might be explained by different availability of SUVH2 or SUVH9 at different developmental stages as well as by possible intensity changes of the histone marks themselves during development. While for example chromocenters show highly condensed H3K9me2 and H3K27me2 signals in nuclei of mature leaves, these signals become successively decondensed in nuclei of senescent leaves (Ay et al. 2009).

**Fig. 6:**
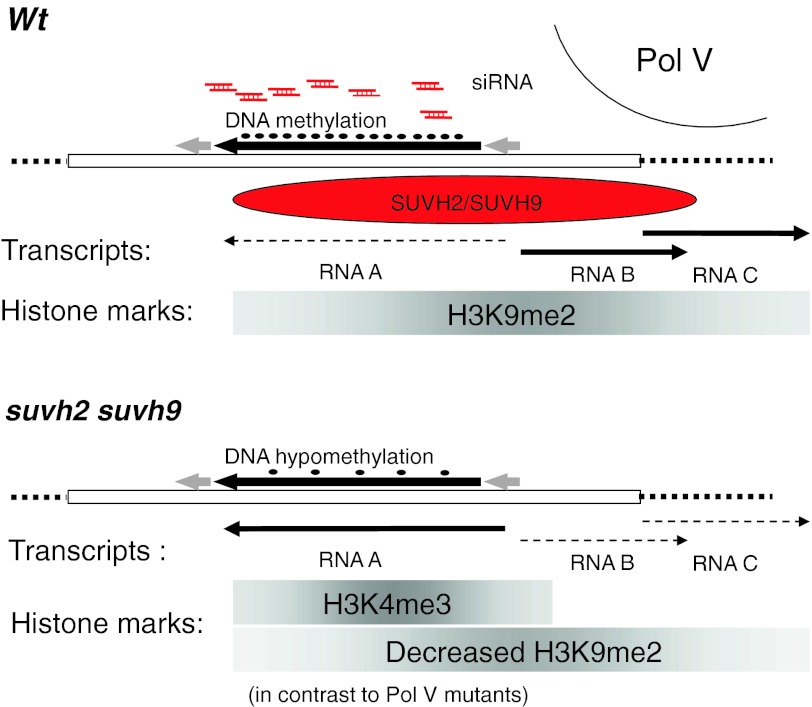
Model for SUVH2 and SUVH9 mediated regulation of *AtSN1.* Schematic overview of the epigenetic status of *AtSN1* in wild type plants (*upper part*) and *suvh2* *suvh9* mutant plants (*lower part*) based on the present study. In the wild type (*Wt*) the *AtSN1* region (*bold black arrow*, with *grey arrows* indicating the LTR borders) is in a transcriptional silenced state in the presence of both histone methyltransferases SUVH2 and SUVH9. The region undergoing RdDM shows the typical hallmarks of TGS, the presence of *AtSN1* A derived 24nt siRNA (*red*) and DNA methylation (*black dots*). The *AtSN1* derived, Pol III dependent transcript A (*dotted arrow*) is silenced, while Pol V dependent transcripts B and C (*black arrows*) can be detected. The transcript A is covering the internal region and the *boxes A* and *B*). The most prominent detected histone modification associated with the complete analyzed *AtSN1* region is histone H3K9me2 (*grey bar*). In the absence of SUVH2 and SUVH9 (*suvh2 suvh9*), silencing is released and the hall marks of RdDM siRNAs and DNA methylation are lost. Pol V dependent transcripts B and C (*dotted arrows*) are not detectable anymore, while the silencing of transcript A (RNA A *black arrow*) is released. The complete *AtSN1* region looses the association with histone H3K9me2, while the region of active transcription (homolog to RNA A) gets associated with H3K4me3

SUVH2 and SUVH9 are required for proper regulation of RdDM targets such as *AtSN1* (Fig. 6). When SUVH2 and SUVH9 were absent, only the *AtSN1* region covered by sense transcript A showed an increased association with H3K4me3 concurrent with transcriptional activation. The regions covered by antisense transcripts B and C did not show such a shift to active marks and Pol V-dependent B transcript was not observed. Nevertheless, the repressive mark H3K9me2 was reduced over the entire *AtSN1* sequence. How does this coordinated loss of H3K9me2 happen? We assume that the size of the analyzed *AtSN1* region plays an important role. As the size of the region corresponding to transcript A correlates approximately to one nucleosome, compact and “locked” structures involving several nucleosomes cannot be established. Such lack of condensation might keep the *AtSN1* region in the state of intermediate heterochromatin (Habu et al. 2006). Such intermediate heterochromatin could provide the basis for SET domain protein involvement in silencing. In the absence of SUVH2 and SUVH9, recruitment of the Pol V complex might be impaired, either by the lack of the SET domain proteins themselves with their ability to bind to methylated DNA, or by the lack of a not yet identified histone modification mediated by SUVH2 and SUVH9. The absence of the SET domain proteins or the histone modification mediated by them could then lead to released TGS at the *AtSN1* region A accompanied by loss of H3K9me2 and DNA methylation. The absence of Pol V might also allow the recruitment of Pol III resulting in formation of transcript A. The specific association of H3K4me3 with the region covered by transcript A within the analyzed region that lost H3K9me2 implies a hierarchy of these modifications. H3K4me3 modification, associated with active transcription, can only be set when the silencing H3K9me2 is absent. This model is supported by the alteration of histone modifications at the *AtSN1* locus observed in *suvh2* *suvh9* mutants which was not described for Pol V deficient plants (Wierzbicki et al. 2008). In addition, *suvh9* and, to a lesser extent, *suvh2* single mutants, did not release TGS of the A region despite a significant loss of DNA methylation. Silencing was released only in the *suvh2* *suvh9* double mutant. Thus, SUVH2 and SUVH9 might act via a pathway independent of Pol V and subsequent de novo DNA methylation. We speculate that SUVH9 and SUVH2 might act via a not yet identified histone methyltransferase activity setting a mark required for subsequent methylation of H3K9 and transcriptional silencing of *AtSN1*.

## Electronic supplementary material

Below is the link to the electronic supplementary material.

Supplementary material 1 (PPT 2213 kb)

Supplementary material 2 (PPT 3226 kb)

Supplementary material 3 (DOCX 20 kb)
