# Supplementary material for: Developmentally non-redundant SET domain proteins SUVH2 and SUVH9 are required for transcriptional gene silencing in Arabidopsis thaliana
Source: Plant Mol Biol. 2012 Jun 6;79(6):623–33. doi: 10.1007/s11103-012-9934-x (PMC3402665; doi:10.1007/s11103-012-9934-x)
Supplement: Supplementary file 1 — Supplementary material 1 (PPT 2213 kb) [file 11103_2012_9934_MOESM1_ESM.ppt]

## Slide 1
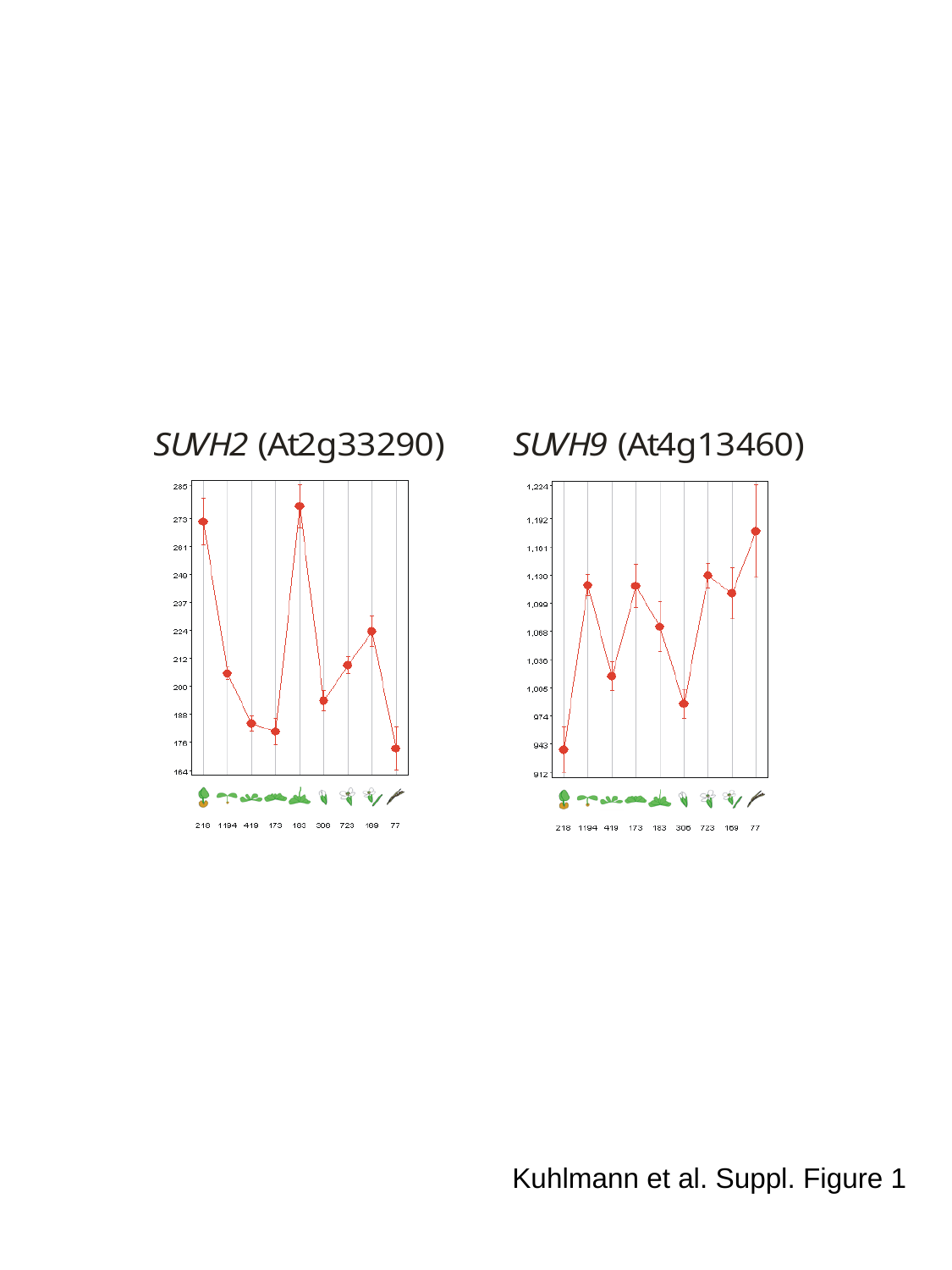

Kuhlmann et al. Suppl. Figure 1

## Slide 2
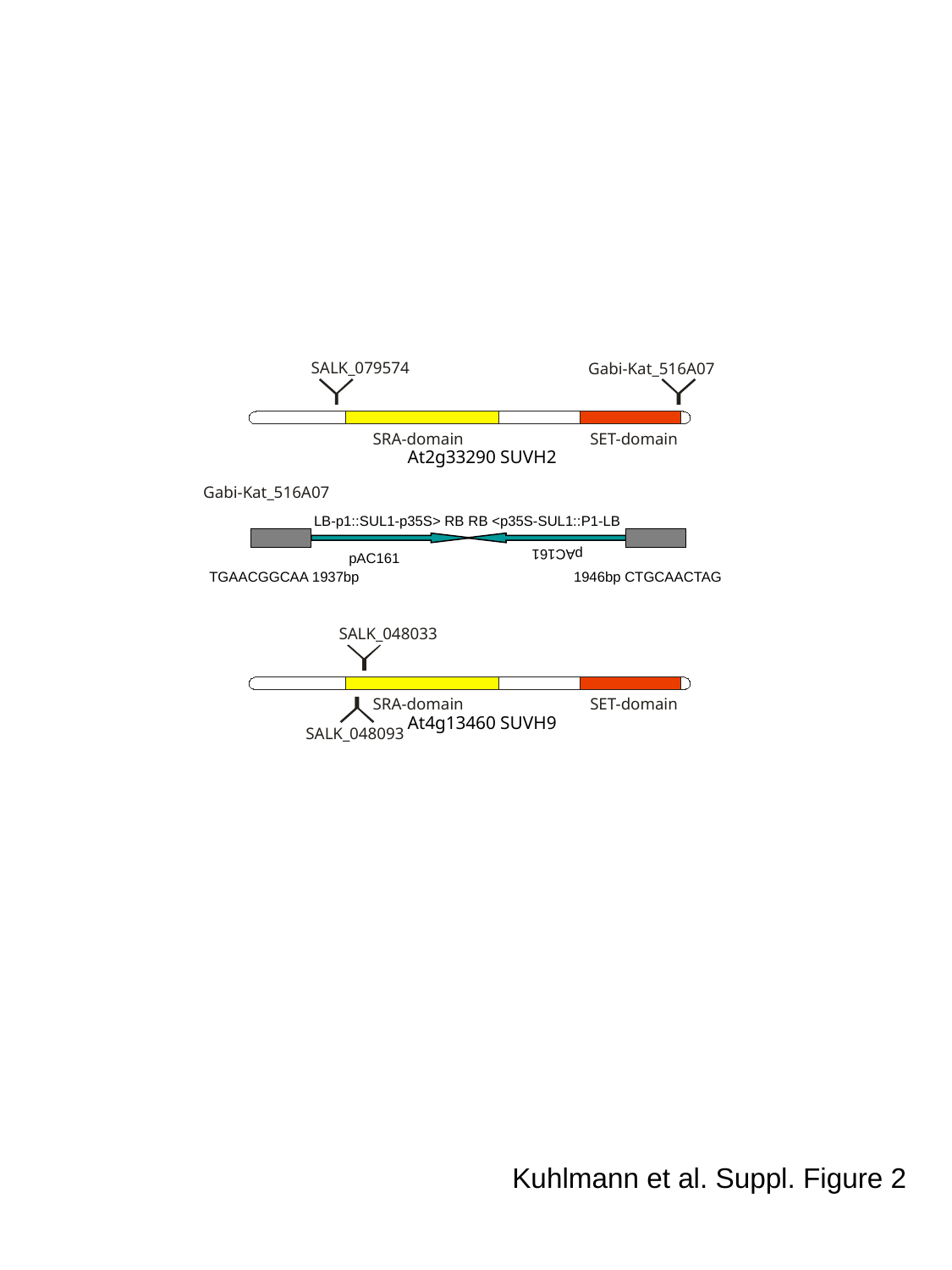

SALK_079574
Gabi-Kat_516A07
SRA-domain
SET-domain
At2g33290 SUVH2
Gabi-Kat_516A07
LB-p1::SUL1-p35S> RB RB <p35S-SUL1::P1-LB
pAC161
pAC161
TGAACGGCAA 1937bp
1946bp CTGCAACTAG
SALK_048033
SRA-domain
SET-domain
At4g13460 SUVH9
SALK_048093
Kuhlmann et al. Suppl. Figure 2

## Slide 3
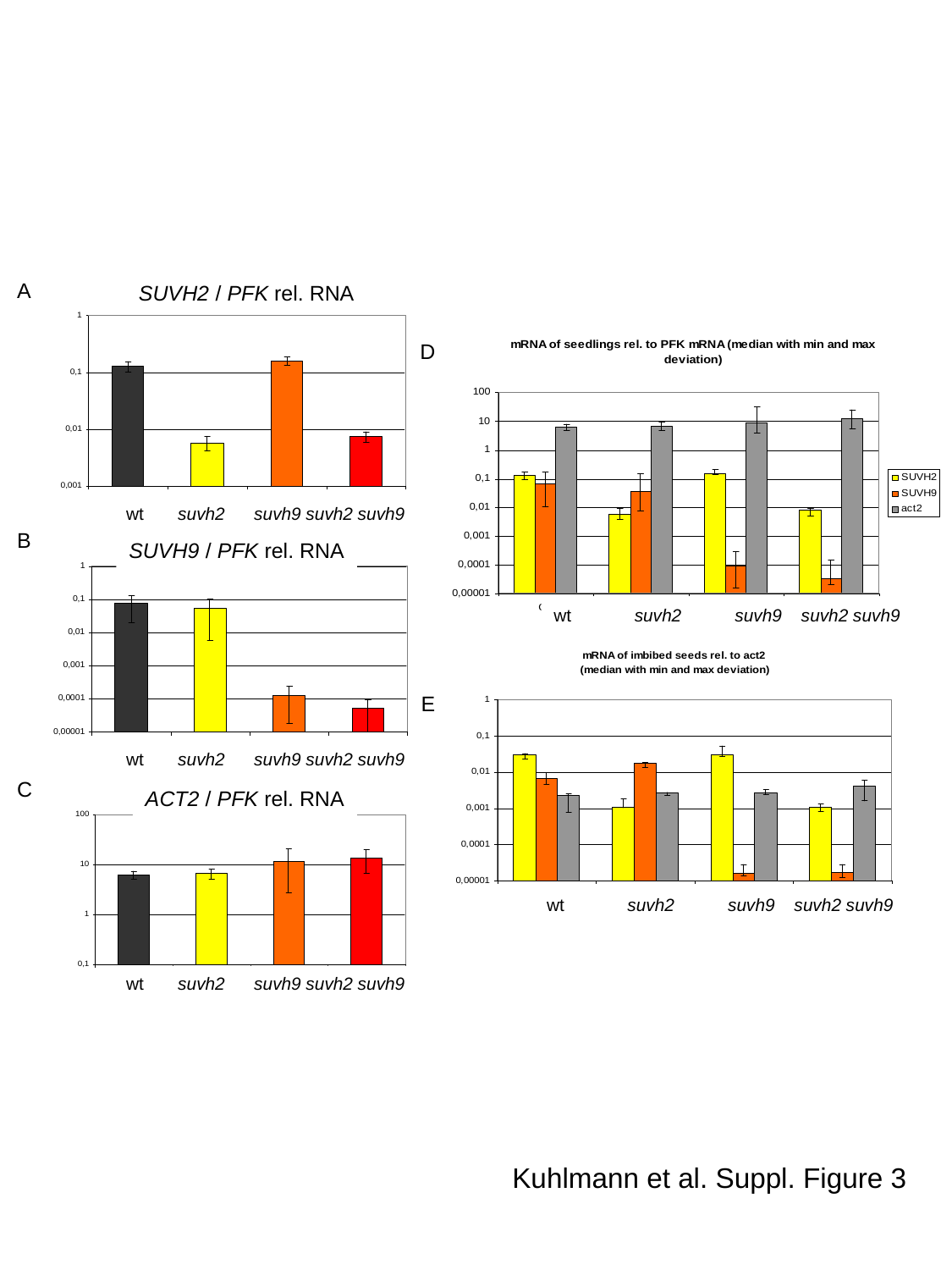

A
SUVH2 / PFK rel. RNA
D
wt suvh2 suvh9 suvh2 suvh9
B
SUVH9 / PFK rel. RNA
wt suvh2 suvh9 suvh2 suvh9
E
wt suvh2 suvh9 suvh2 suvh9
C
ACT2 / PFK rel. RNA
wt suvh2 suvh9 suvh2 suvh9
wt suvh2 suvh9 suvh2 suvh9
Kuhlmann et al. Suppl. Figure 3

## Slide 4
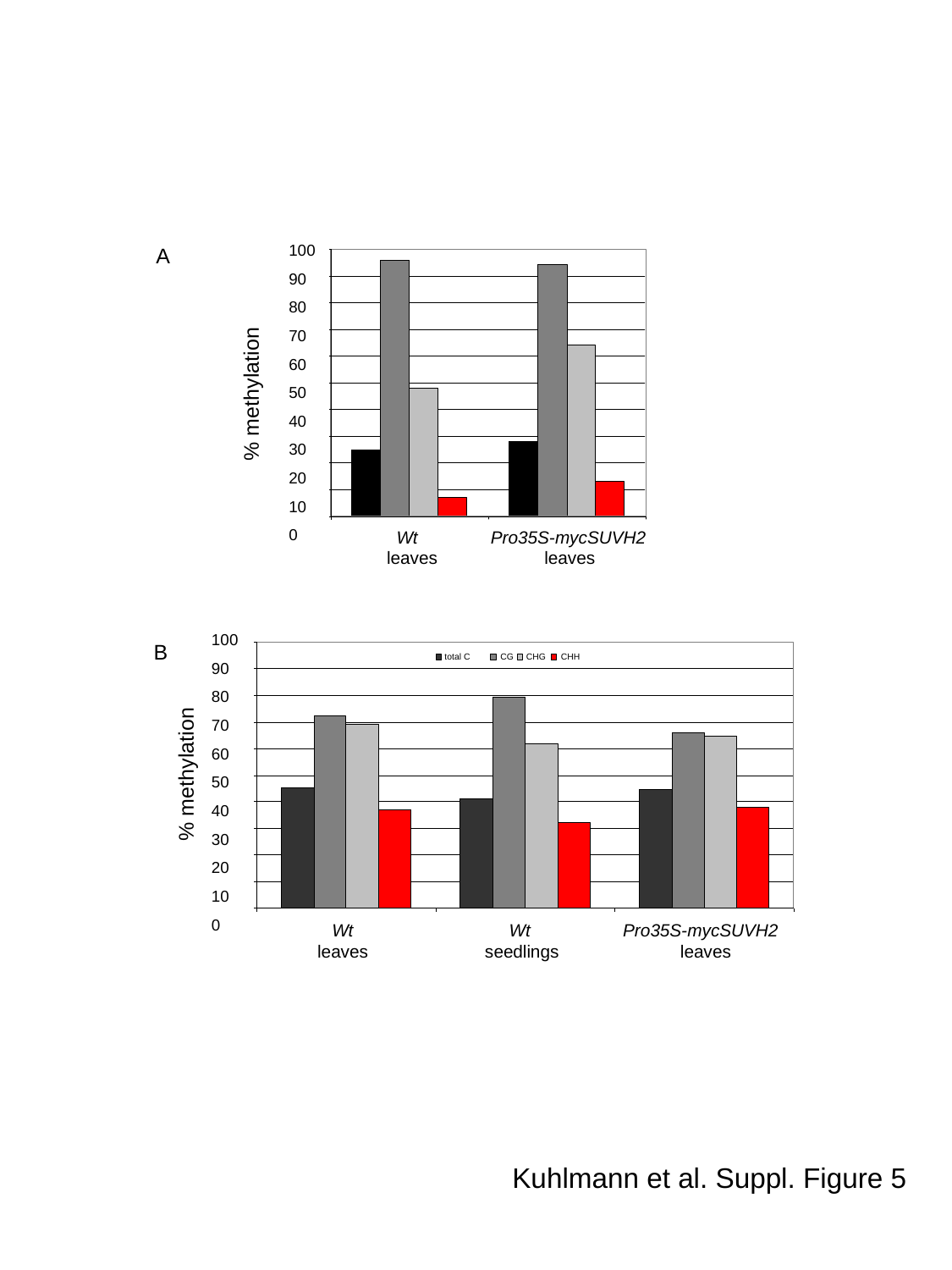

A
100
90
80
70
60
50
40
30
20
10
0
% methylation
 Wt Pro35S-mycSUVH2
 leaves leaves
100
90
80
70
60
50
40
30
20
10
0
B
total C CG CHG CHH
% methylation
 Wt Wt Pro35S-mycSUVH2
 leaves seedlings leaves
Kuhlmann et al. Suppl. Figure 5

## Slide 5
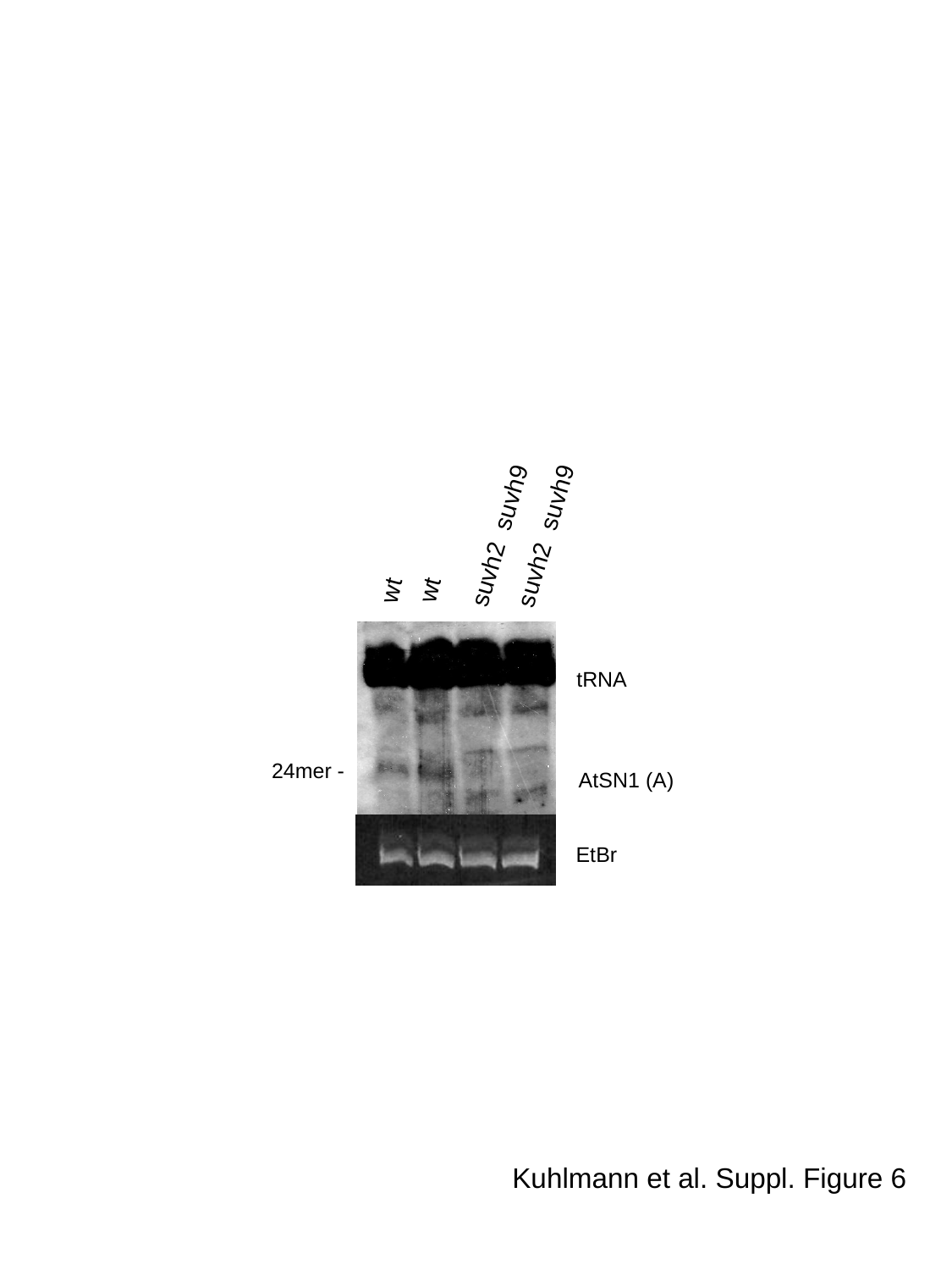

suvh2 suvh9
suvh2 suvh9
wt
wt
tRNA
24mer -
AtSN1 (A)
EtBr
Kuhlmann et al. Suppl. Figure 6

## Slide 6
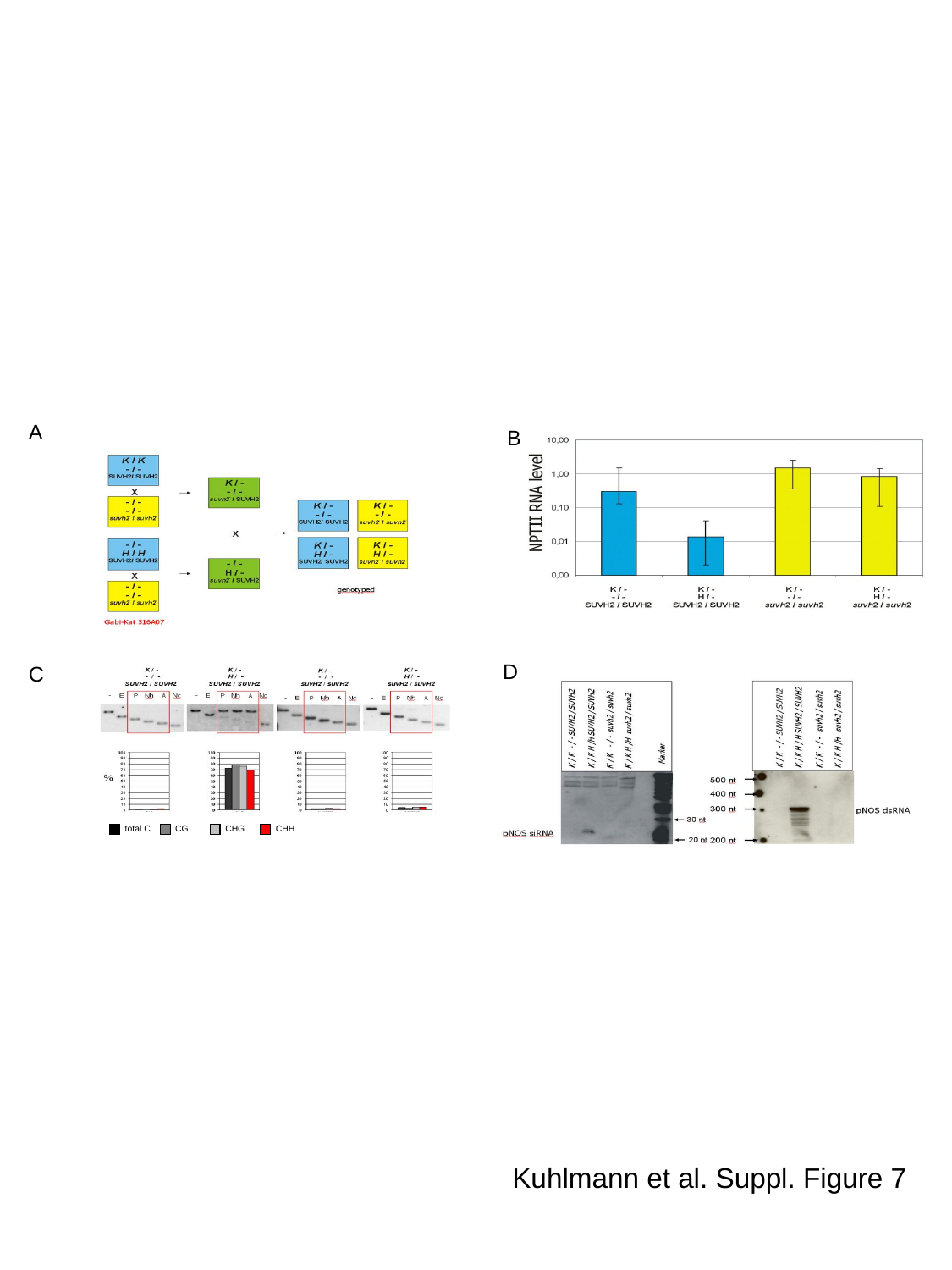

A
B
D
C
total C
CG
CHG
CHH
Kuhlmann et al. Suppl. Figure 7

## Slide 7
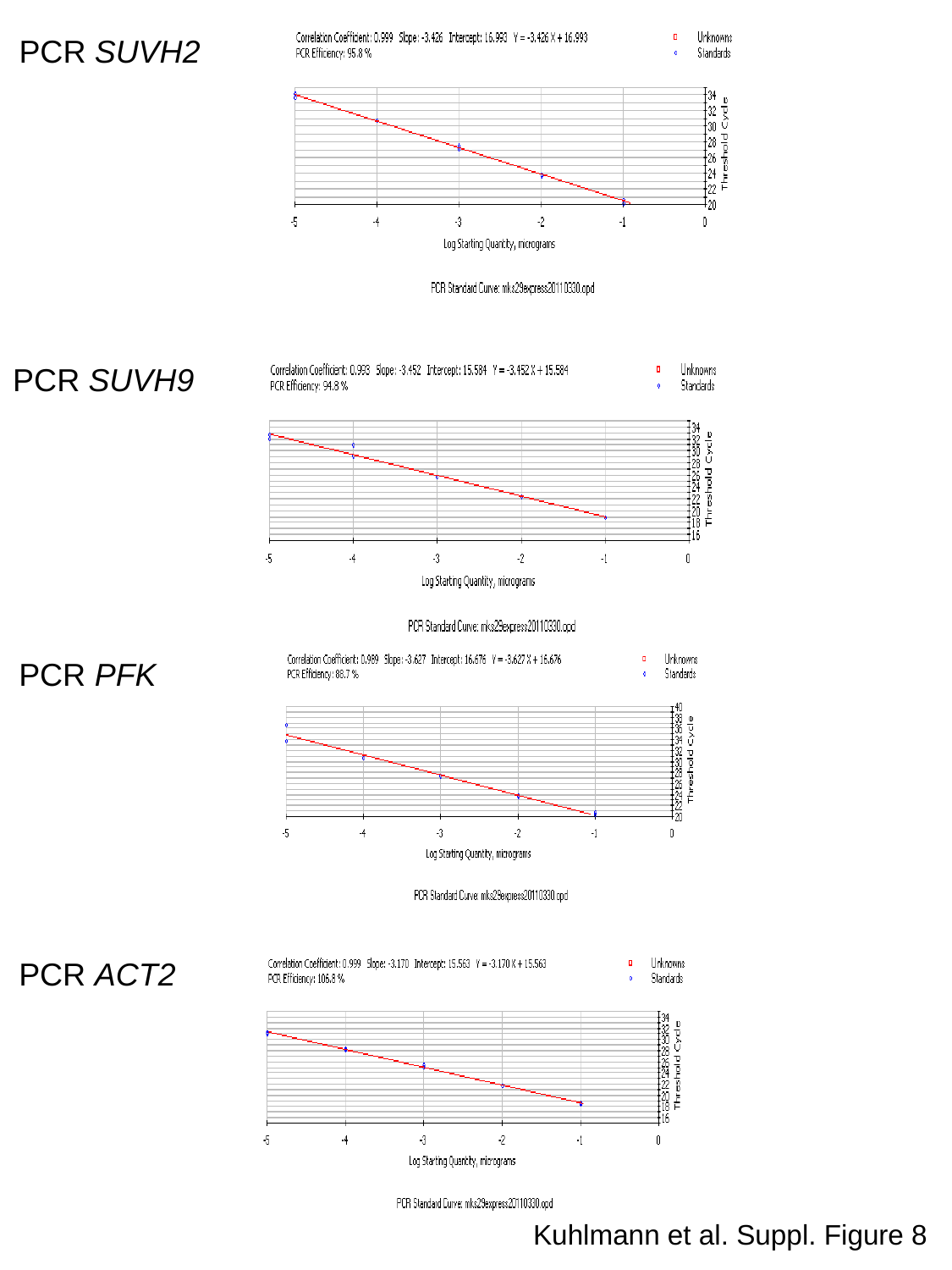

PCR SUVH2
PCR SUVH9
PCR PFK
PCR ACT2
Kuhlmann et al. Suppl. Figure 8

## Slide 8
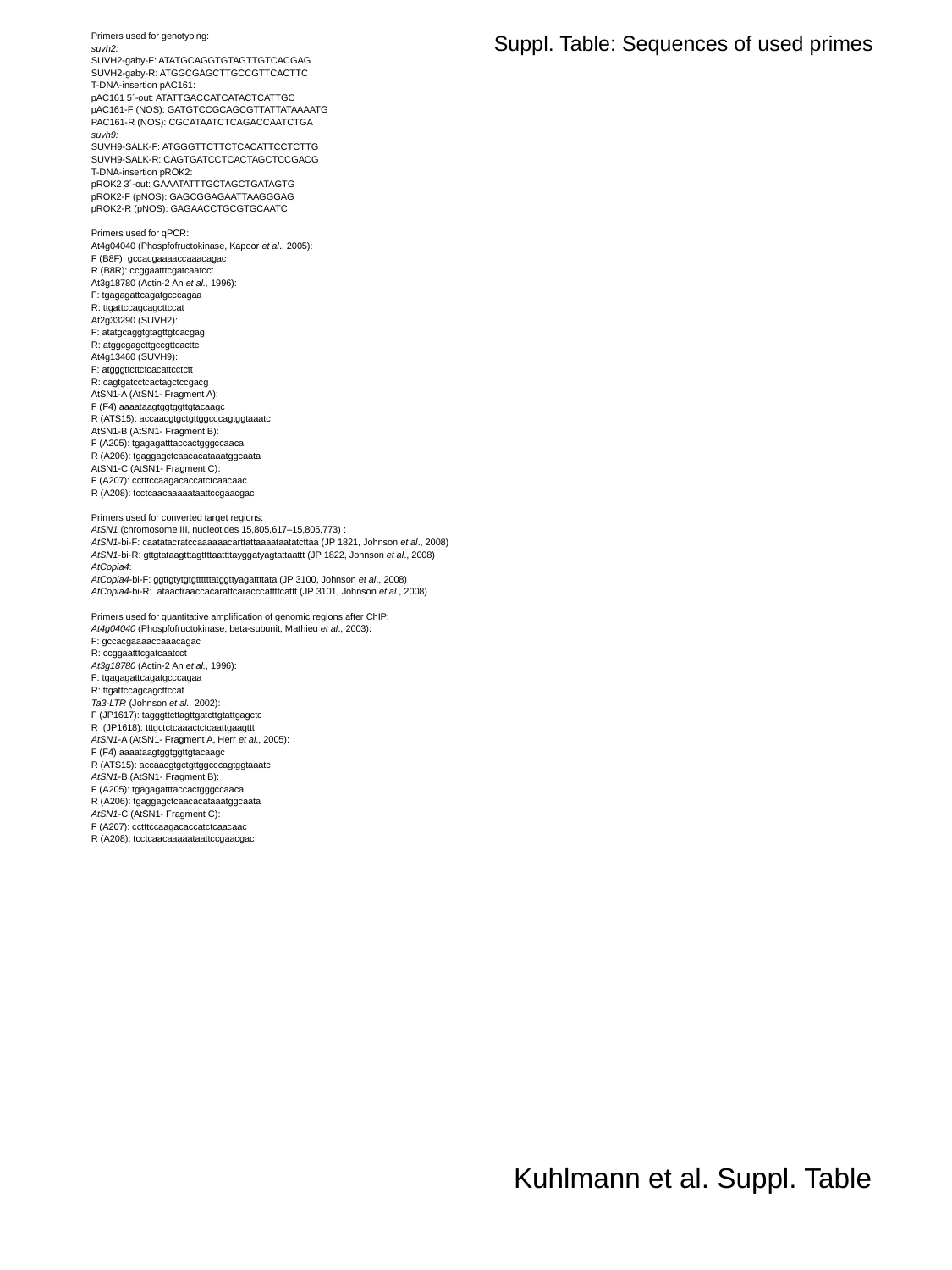

# Suppl. Table: Sequences of used primes
Primers used for genotyping:
suvh2:
SUVH2-gaby-F: ATATGCAGGTGTAGTTGTCACGAG
SUVH2-gaby-R: ATGGCGAGCTTGCCGTTCACTTC
T-DNA-insertion pAC161:
pAC161 5´-out: ATATTGACCATCATACTCATTGC
pAC161-F (NOS): GATGTCCGCAGCGTTATTATAAAATG
PAC161-R (NOS): CGCATAATCTCAGACCAATCTGA
suvh9:
SUVH9-SALK-F: ATGGGTTCTTCTCACATTCCTCTTG
SUVH9-SALK-R: CAGTGATCCTCACTAGCTCCGACG
T-DNA-insertion pROK2:
pROK2 3´-out: GAAATATTTGCTAGCTGATAGTG
pROK2-F (pNOS): GAGCGGAGAATTAAGGGAG
pROK2-R (pNOS): GAGAACCTGCGTGCAATC
Primers used for qPCR:
At4g04040 (Phospfofructokinase, Kapoor et al., 2005):
F (B8F): gccacgaaaaccaaacagac
R (B8R): ccggaatttcgatcaatcct
At3g18780 (Actin-2 An et al., 1996):
F: tgagagattcagatgcccagaa
R: ttgattccagcagcttccat
At2g33290 (SUVH2):
F: atatgcaggtgtagttgtcacgag
R: atggcgagcttgccgttcacttc
At4g13460 (SUVH9):
F: atgggttcttctcacattcctctt
R: cagtgatcctcactagctccgacg
AtSN1-A (AtSN1- Fragment A):
F (F4) aaaataagtggtggttgtacaagc
R (ATS15): accaacgtgctgttggcccagtggtaaatc
AtSN1-B (AtSN1- Fragment B):
F (A205): tgagagatttaccactgggccaaca
R (A206): tgaggagctcaacacataaatggcaata
AtSN1-C (AtSN1- Fragment C):
F (A207): cctttccaagacaccatctcaacaac
R (A208): tcctcaacaaaaataattccgaacgac
Primers used for converted target regions:
AtSN1 (chromosome III, nucleotides 15,805,617–15,805,773) :
AtSN1-bi-F: caatatacratccaaaaaacarttattaaaataatatcttaa (JP 1821, Johnson et al., 2008)
AtSN1-bi-R: gttgtataagtttagttttaattttayggatyagtattaattt (JP 1822, Johnson et al., 2008)
AtCopia4:
AtCopia4-bi-F: ggttgtytgtgttttttatggttyagattttata (JP 3100, Johnson et al., 2008)
AtCopia4-bi-R: ataactraaccacarattcaracccattttcattt (JP 3101, Johnson et al., 2008)
Primers used for quantitative amplification of genomic regions after ChIP:
At4g04040 (Phospfofructokinase, beta-subunit, Mathieu et al., 2003):
F: gccacgaaaaccaaacagac
R: ccggaatttcgatcaatcct
At3g18780 (Actin-2 An et al., 1996):
F: tgagagattcagatgcccagaa
R: ttgattccagcagcttccat
Ta3-LTR (Johnson et al., 2002):
F (JP1617): tagggttcttagttgatcttgtattgagctc
R  (JP1618): tttgctctcaaactctcaattgaagttt
AtSN1-A (AtSN1- Fragment A, Herr et al., 2005):
F (F4) aaaataagtggtggttgtacaagc
R (ATS15): accaacgtgctgttggcccagtggtaaatc
AtSN1-B (AtSN1- Fragment B):
F (A205): tgagagatttaccactgggccaaca
R (A206): tgaggagctcaacacataaatggcaata
AtSN1-C (AtSN1- Fragment C):
F (A207): cctttccaagacaccatctcaacaac
R (A208): tcctcaacaaaaataattccgaacgac
Kuhlmann et al. Suppl. Table
