# Supplementary material for: Developmentally non-redundant SET domain proteins SUVH2 and SUVH9 are required for transcriptional gene silencing in Arabidopsis thaliana
Source: Plant Mol Biol. 2012 Jun 6;79(6):623–33. doi: 10.1007/s11103-012-9934-x (PMC3402665; doi:10.1007/s11103-012-9934-x)
Supplement: Supplementary file 2 — Supplementary material 2 (PPT 3226 kb) [file 11103_2012_9934_MOESM2_ESM.ppt]

## Slide 1
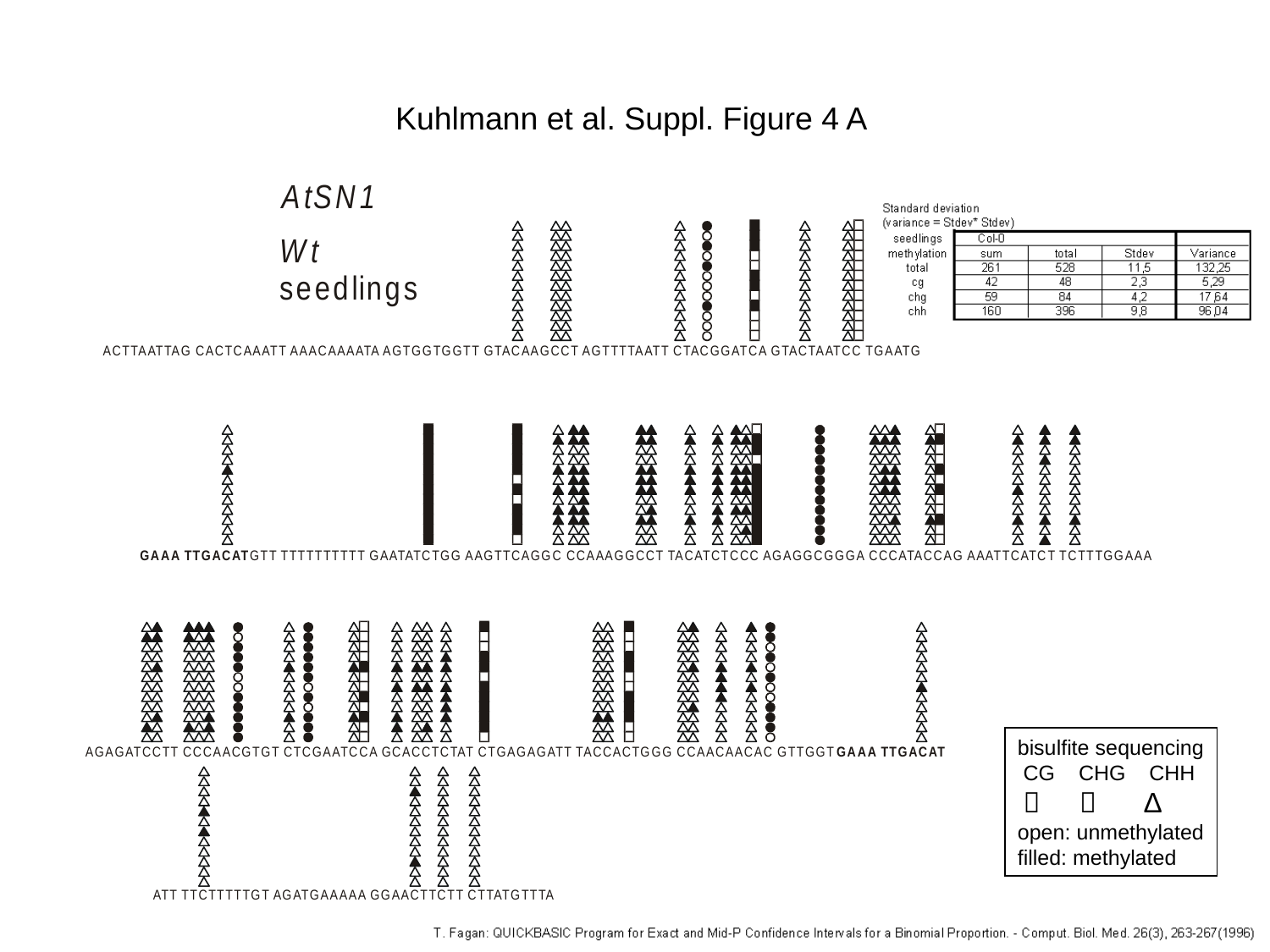

# Kuhlmann et al. Suppl. Figure 4 A
bisulfite sequencing
 CG CHG CHH
   Δ
open: unmethylated
filled: methylated

## Slide 2
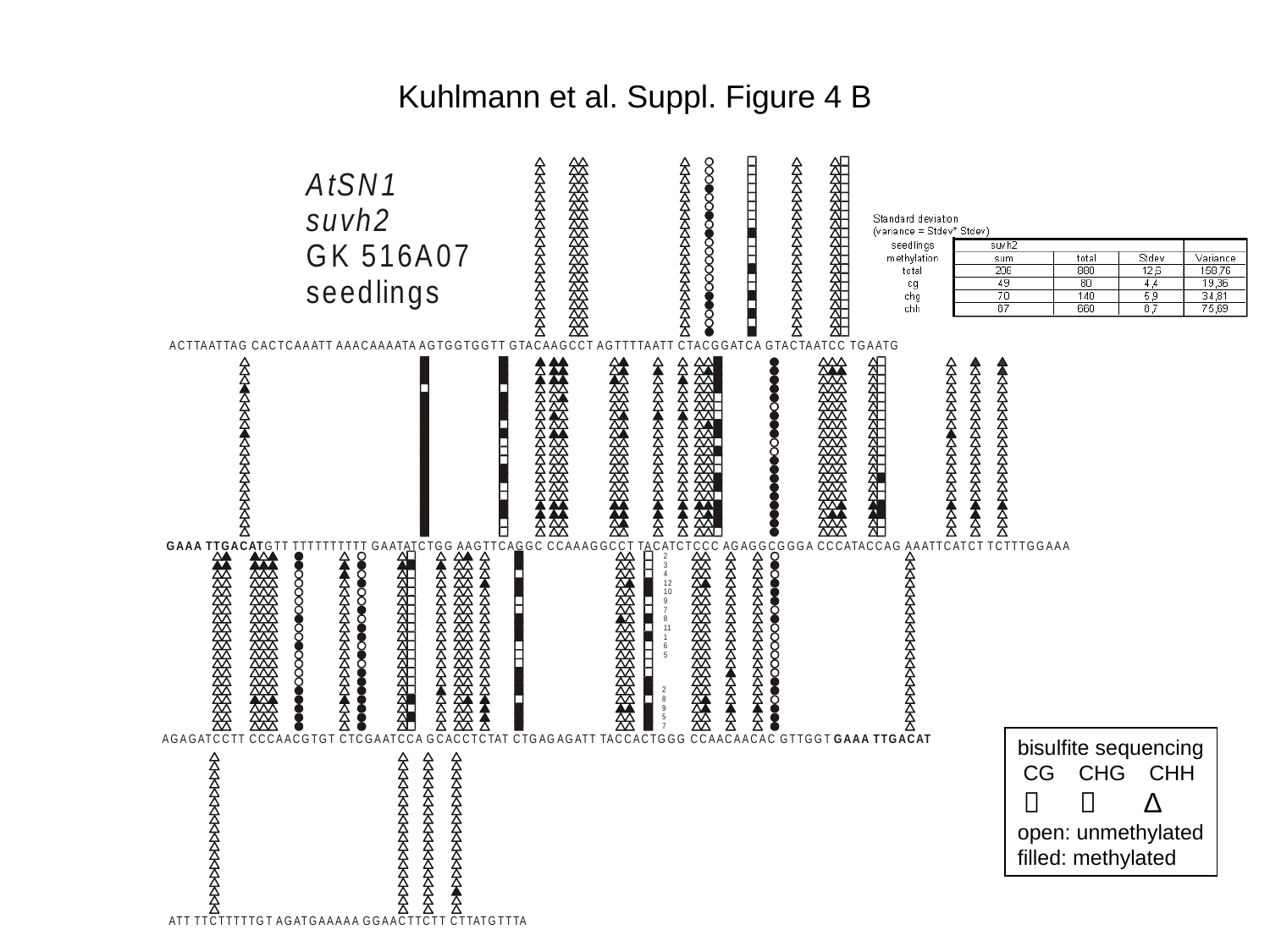

# Kuhlmann et al. Suppl. Figure 4 B
bisulfite sequencing
 CG CHG CHH
   Δ
open: unmethylated
filled: methylated

## Slide 3
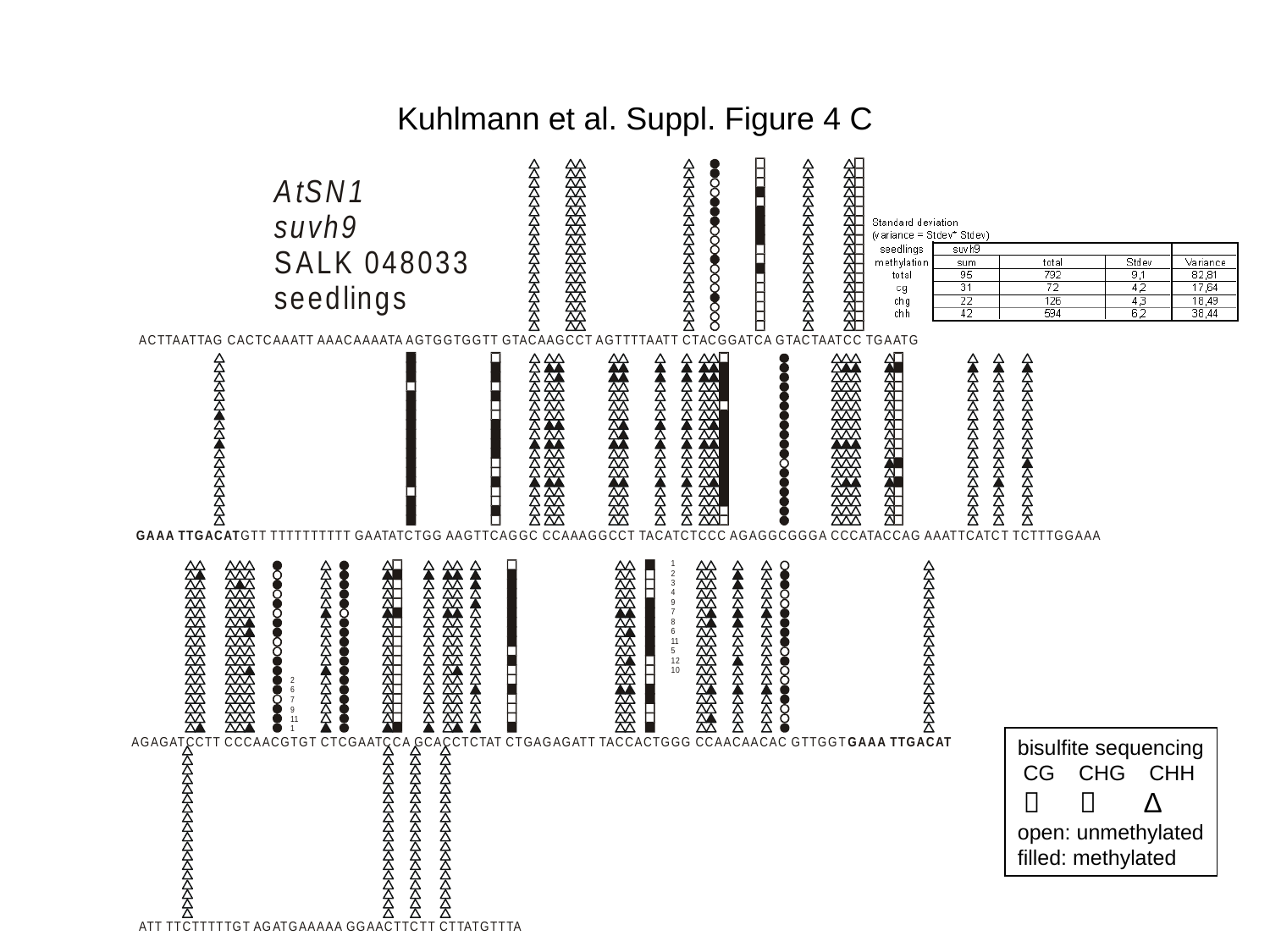

# Kuhlmann et al. Suppl. Figure 4 C
bisulfite sequencing
 CG CHG CHH
   Δ
open: unmethylated
filled: methylated

## Slide 4
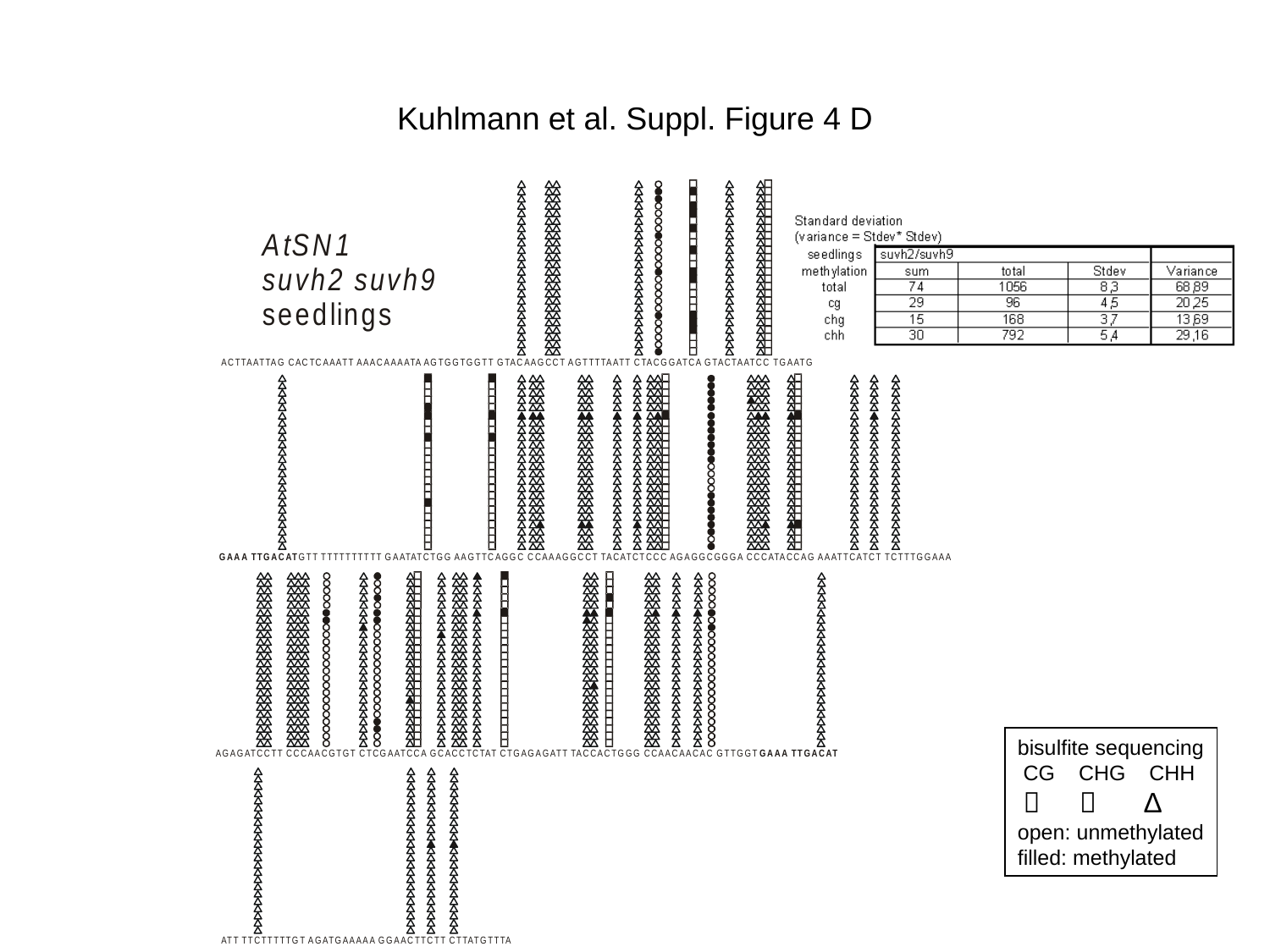

# Kuhlmann et al. Suppl. Figure 4 D
bisulfite sequencing
 CG CHG CHH
   Δ
open: unmethylated
filled: methylated

## Slide 5
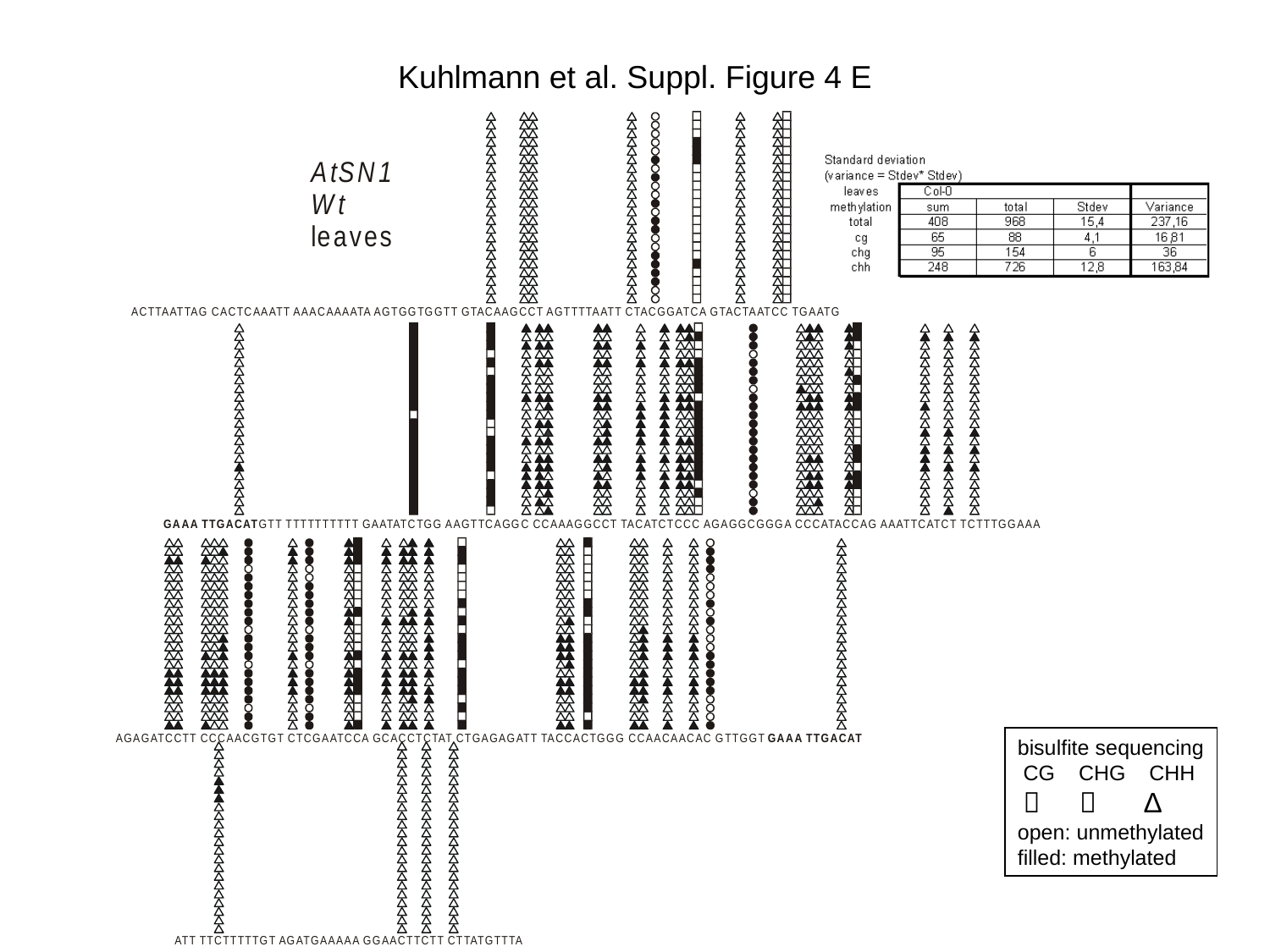

# Kuhlmann et al. Suppl. Figure 4 E
bisulfite sequencing
 CG CHG CHH
   Δ
open: unmethylated
filled: methylated

## Slide 6
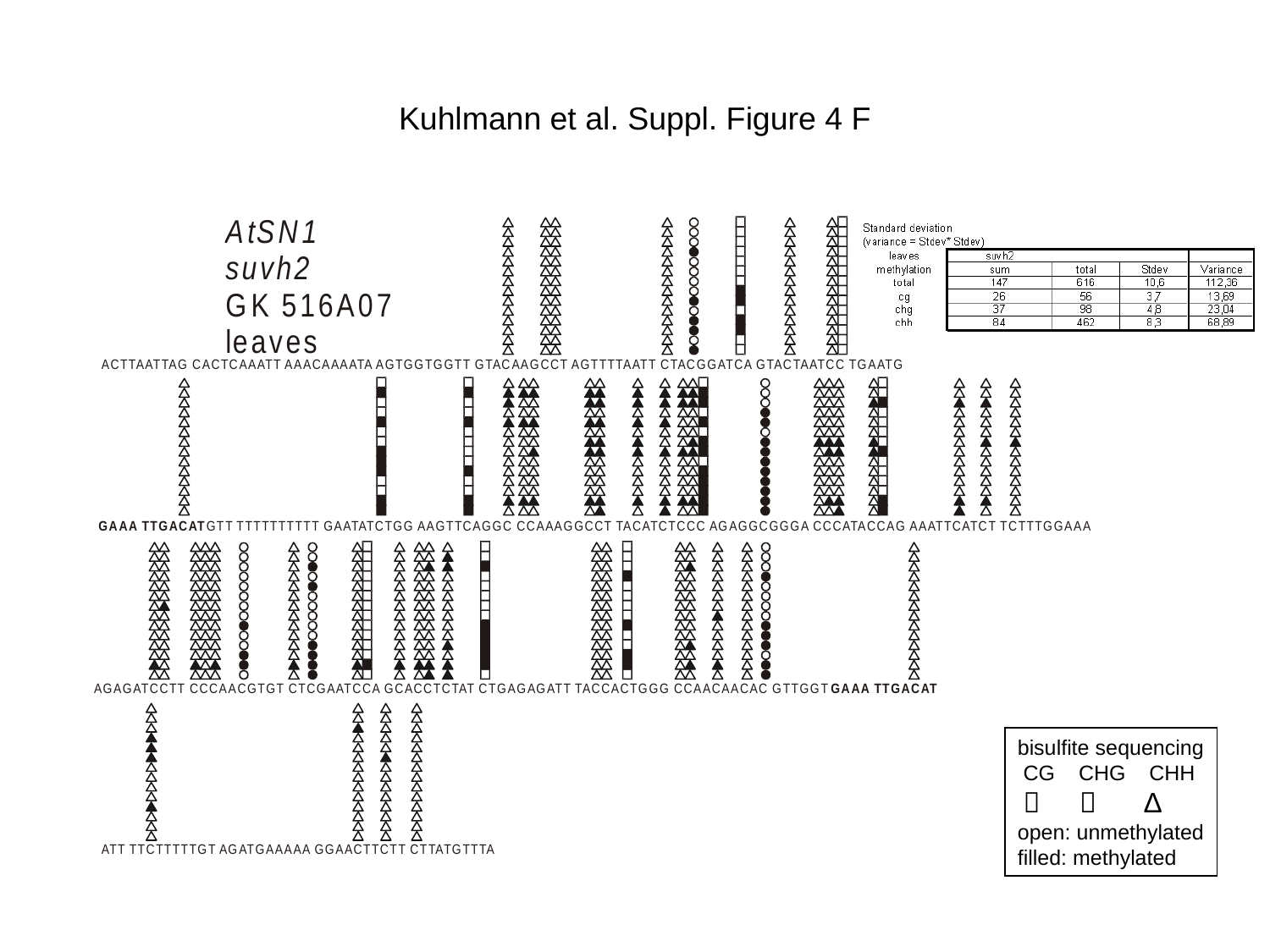

# Kuhlmann et al. Suppl. Figure 4 F
bisulfite sequencing
 CG CHG CHH
   Δ
open: unmethylated
filled: methylated

## Slide 7
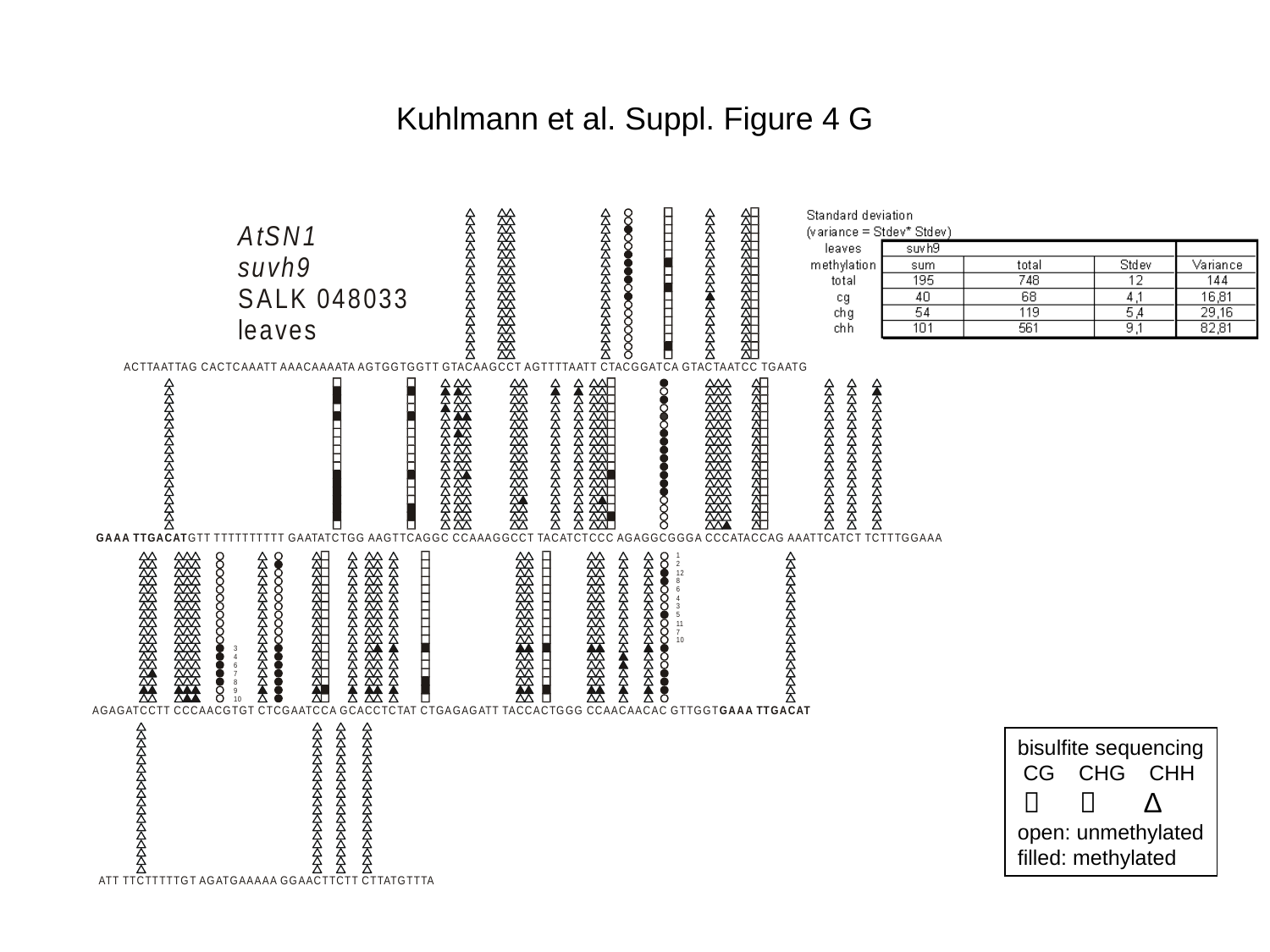

# Kuhlmann et al. Suppl. Figure 4 G
bisulfite sequencing
 CG CHG CHH
   Δ
open: unmethylated
filled: methylated

## Slide 8
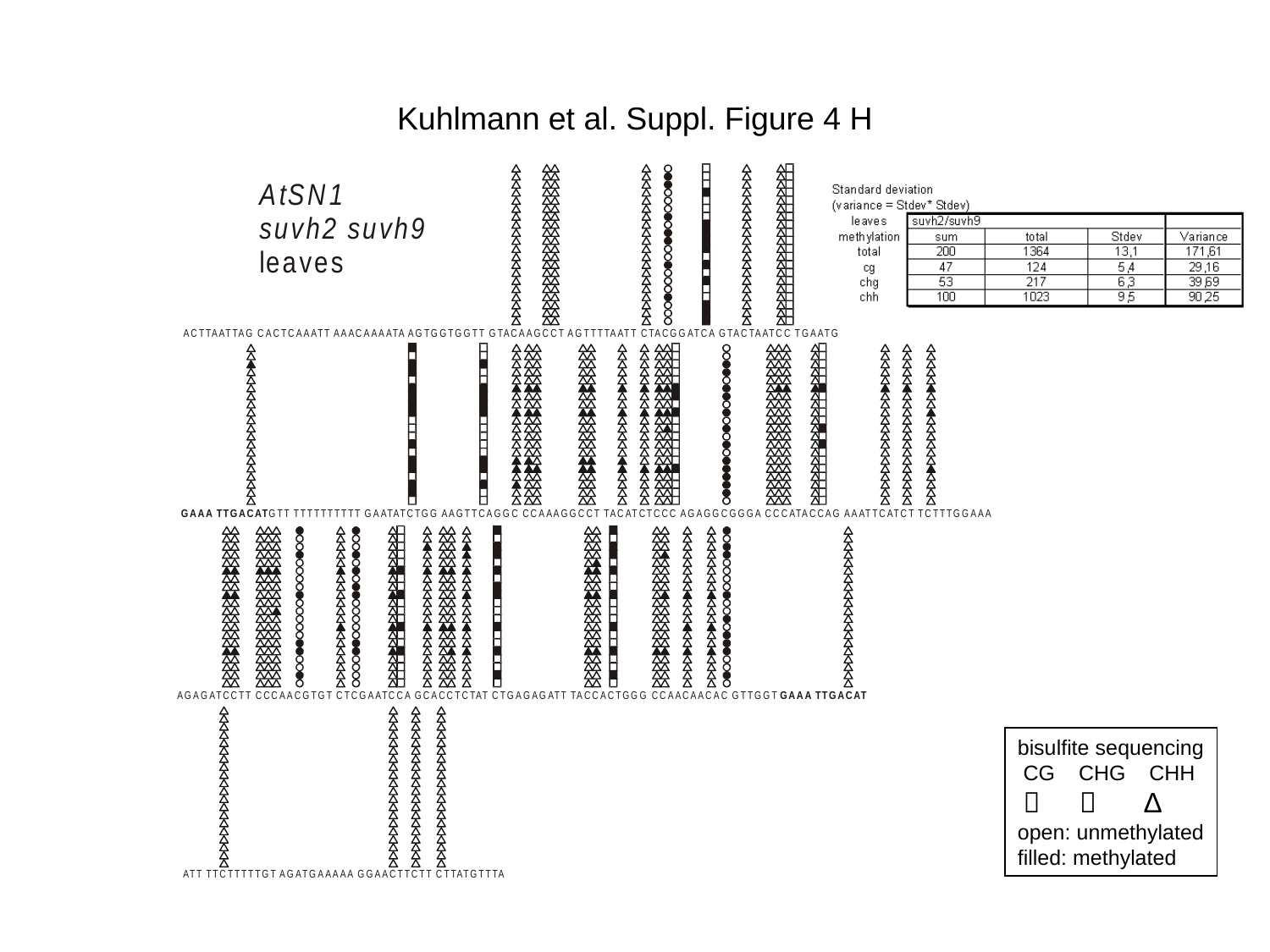

# Kuhlmann et al. Suppl. Figure 4 H
bisulfite sequencing
 CG CHG CHH
   Δ
open: unmethylated
filled: methylated

## Slide 9
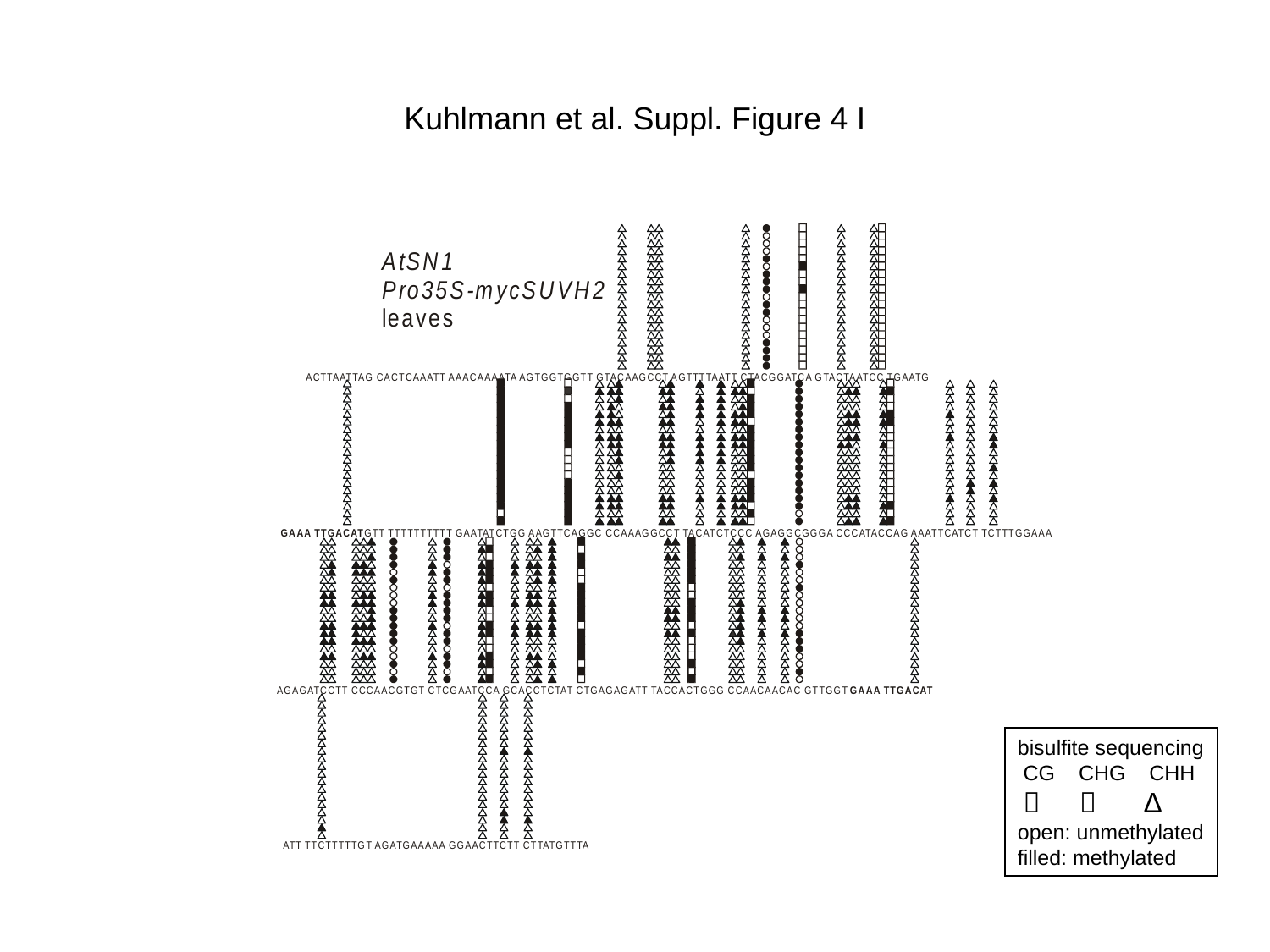

# Kuhlmann et al. Suppl. Figure 4 I
bisulfite sequencing
 CG CHG CHH
   Δ
open: unmethylated
filled: methylated

## Slide 10
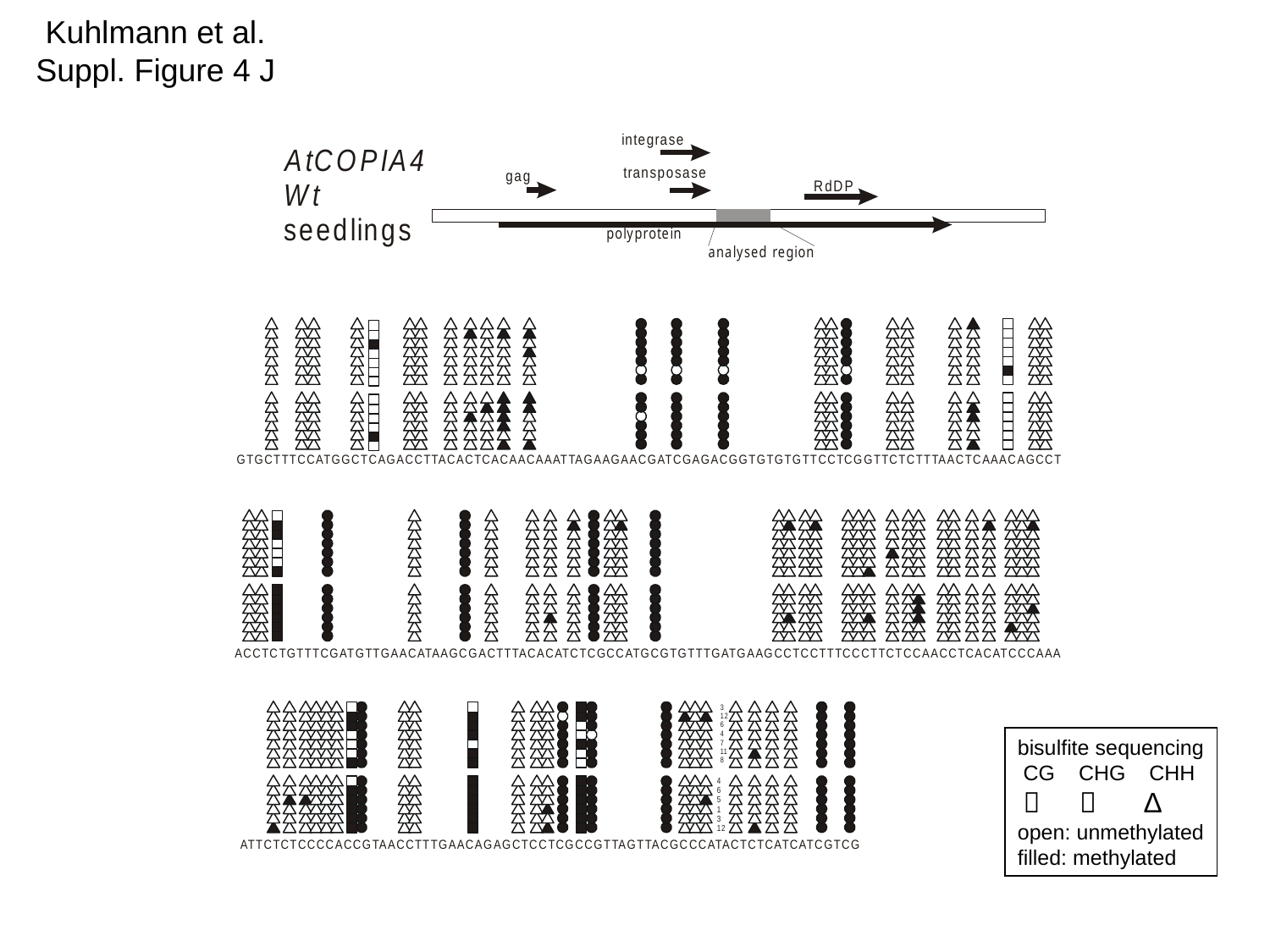

# Kuhlmann et al. Suppl. Figure 4 J
bisulfite sequencing
 CG CHG CHH
   Δ
open: unmethylated
filled: methylated

## Slide 11
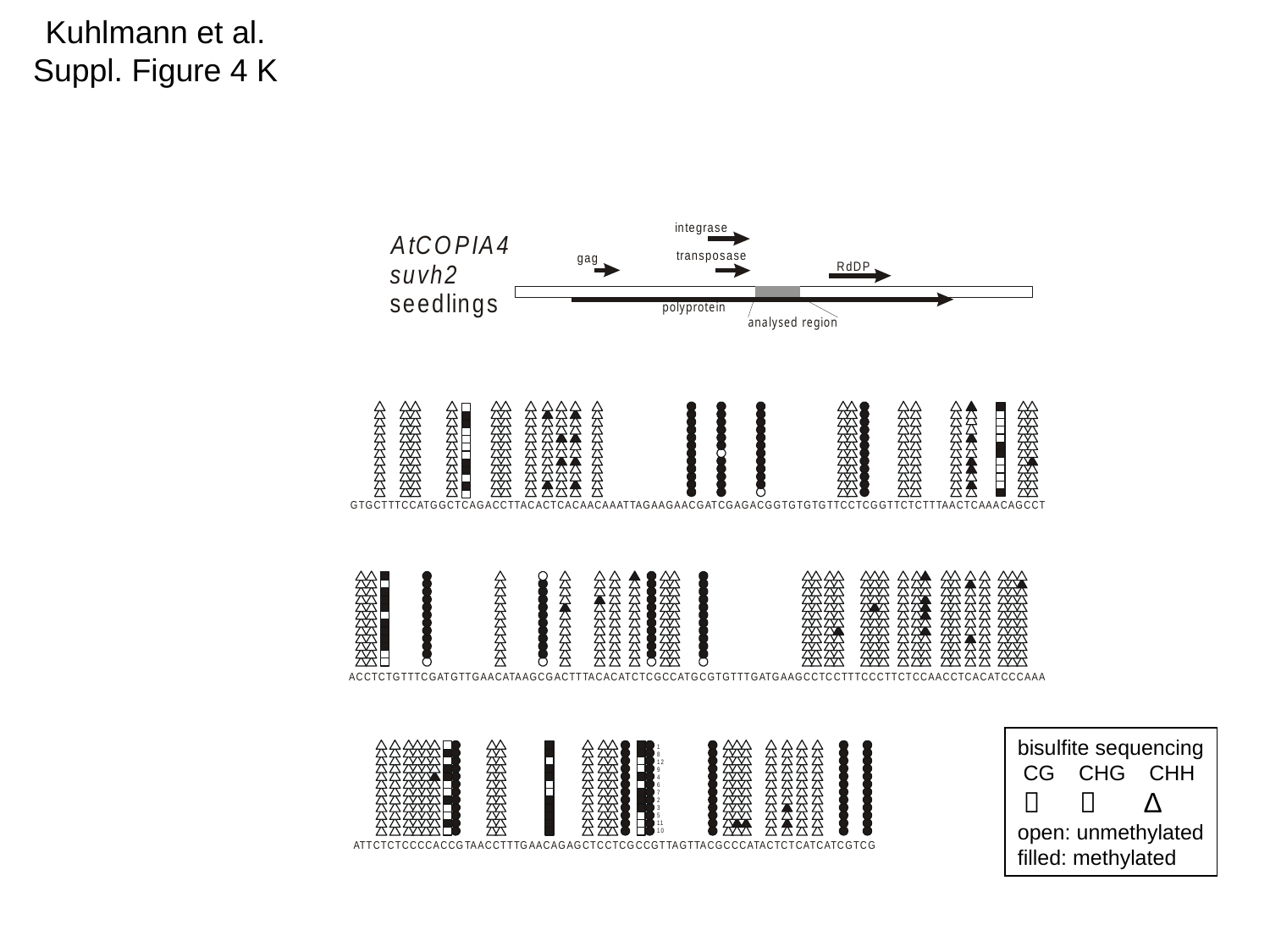

# Kuhlmann et al. Suppl. Figure 4 K
bisulfite sequencing
 CG CHG CHH
   Δ
open: unmethylated
filled: methylated

## Slide 12
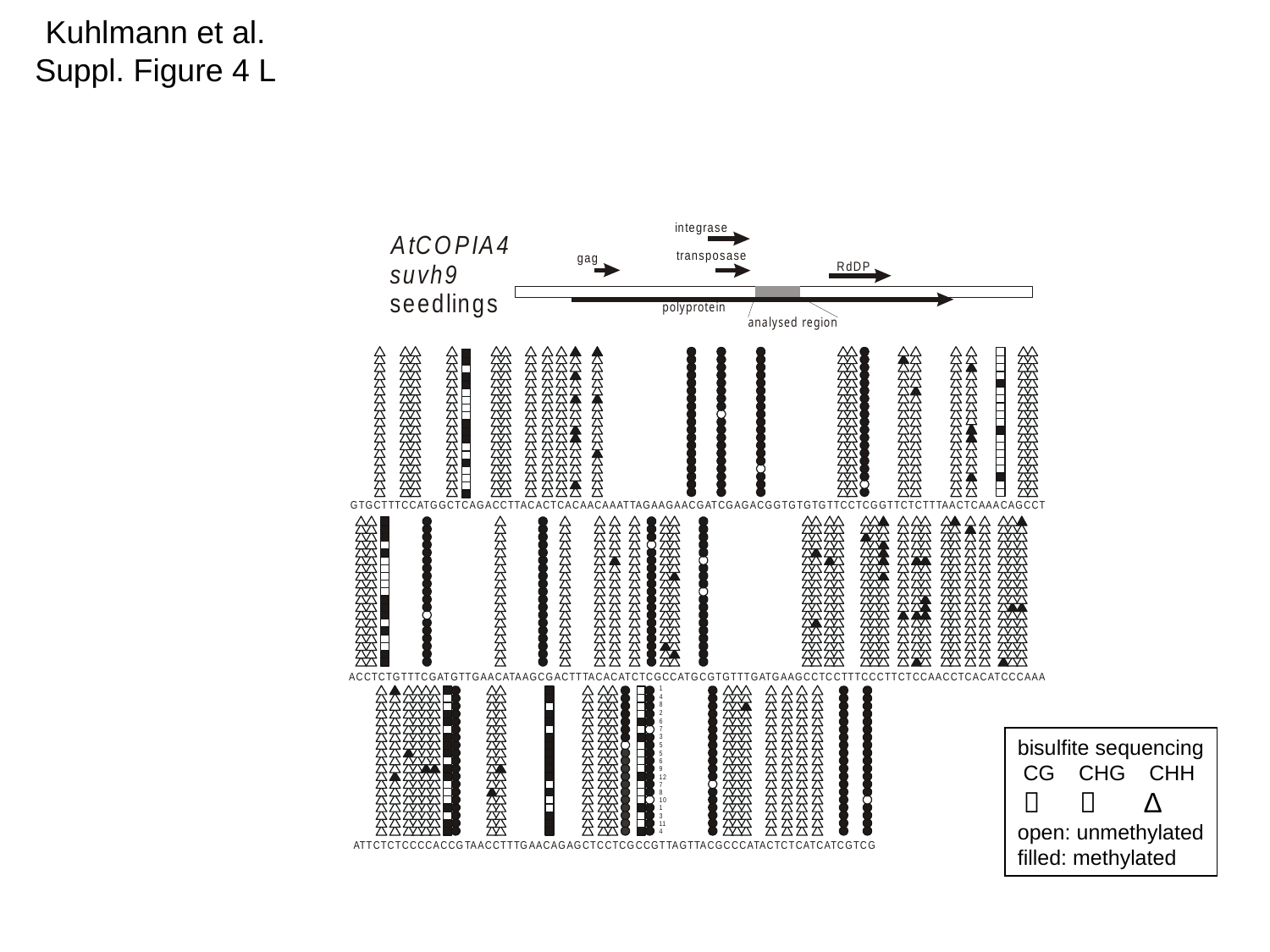

# Kuhlmann et al. Suppl. Figure 4 L
bisulfite sequencing
 CG CHG CHH
   Δ
open: unmethylated
filled: methylated

## Slide 13
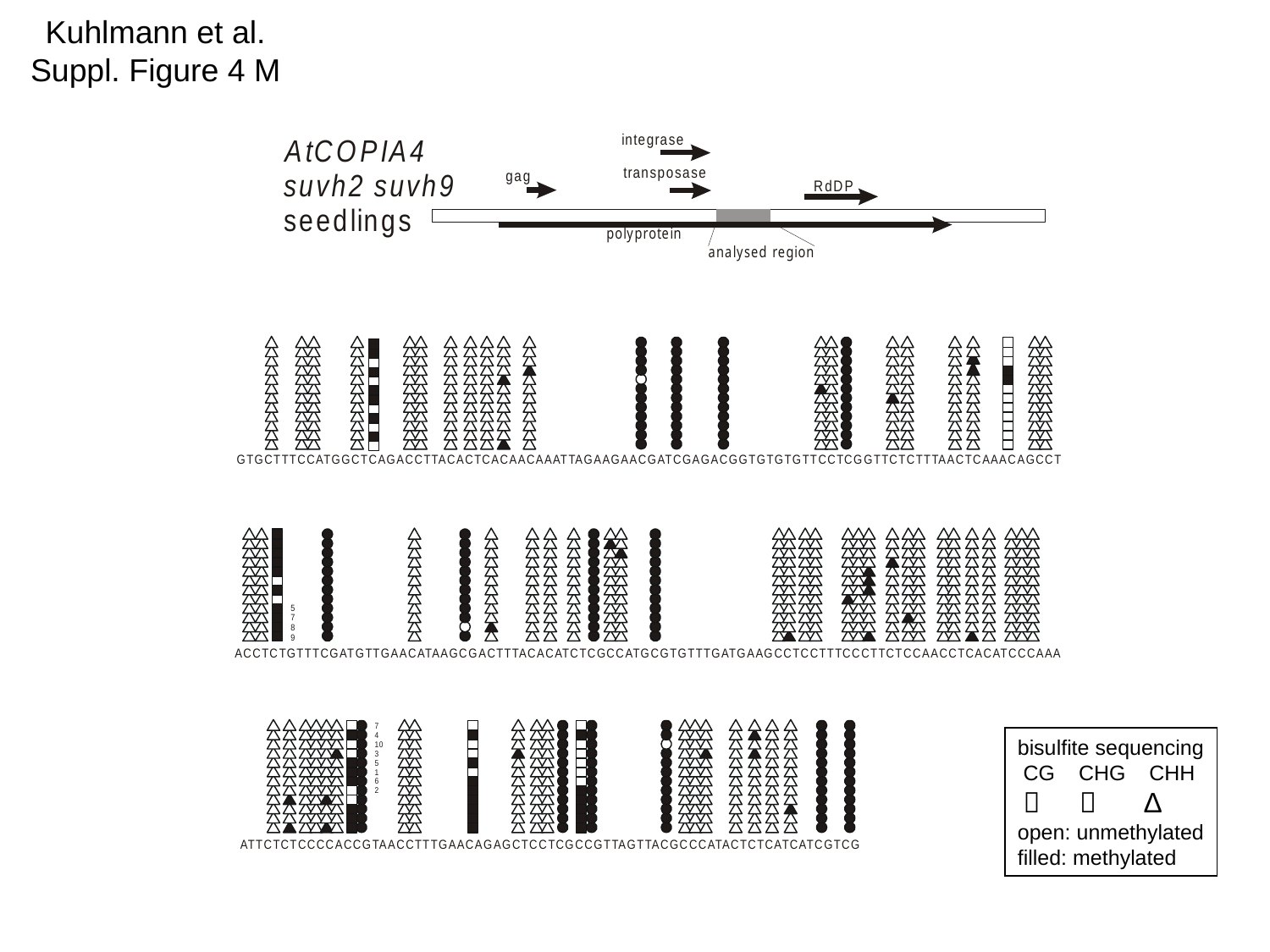

# Kuhlmann et al. Suppl. Figure 4 M
bisulfite sequencing
 CG CHG CHH
   Δ
open: unmethylated
filled: methylated

## Slide 14
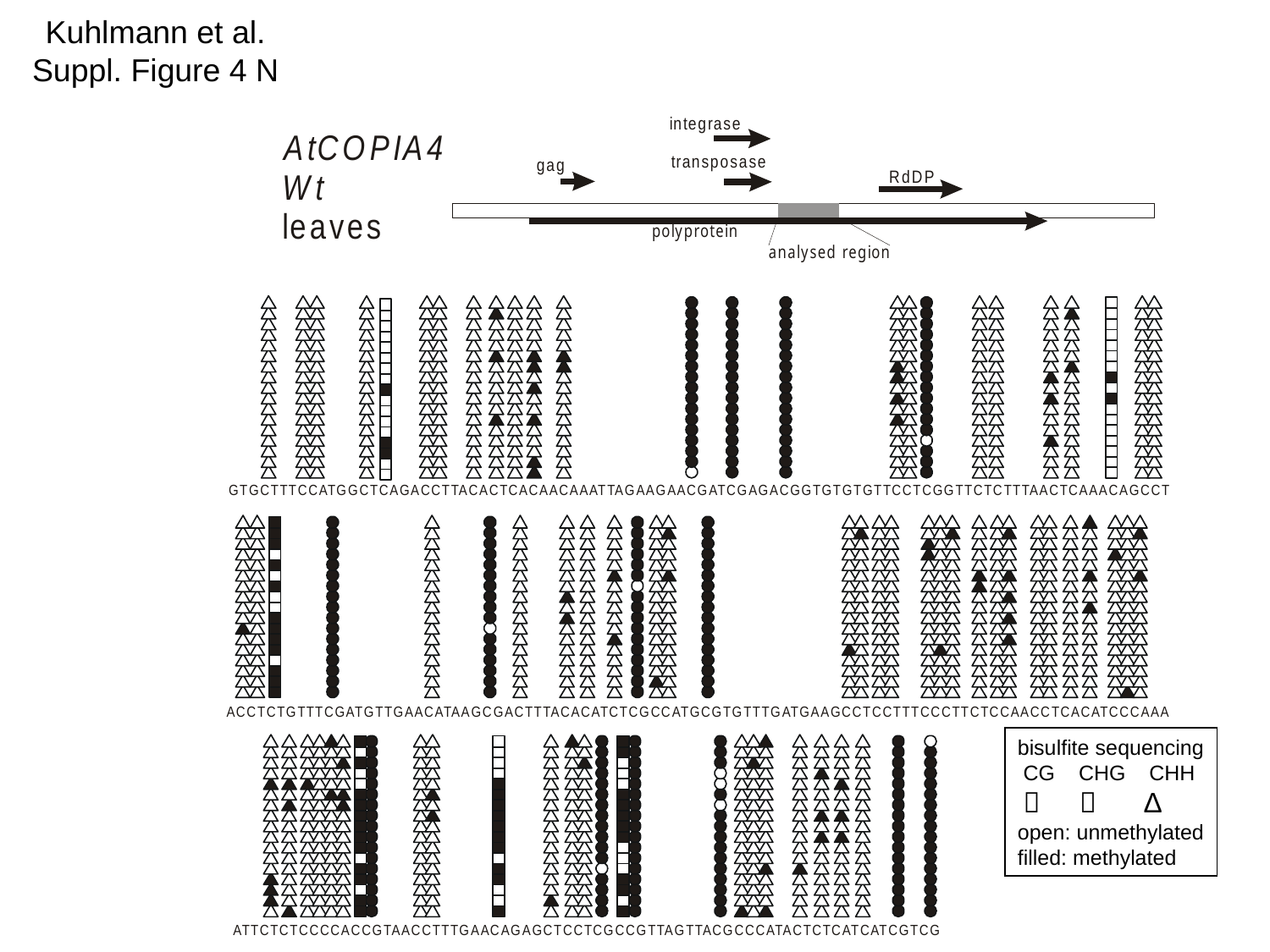

# Kuhlmann et al. Suppl. Figure 4 N
bisulfite sequencing
 CG CHG CHH
   Δ
open: unmethylated
filled: methylated

## Slide 15
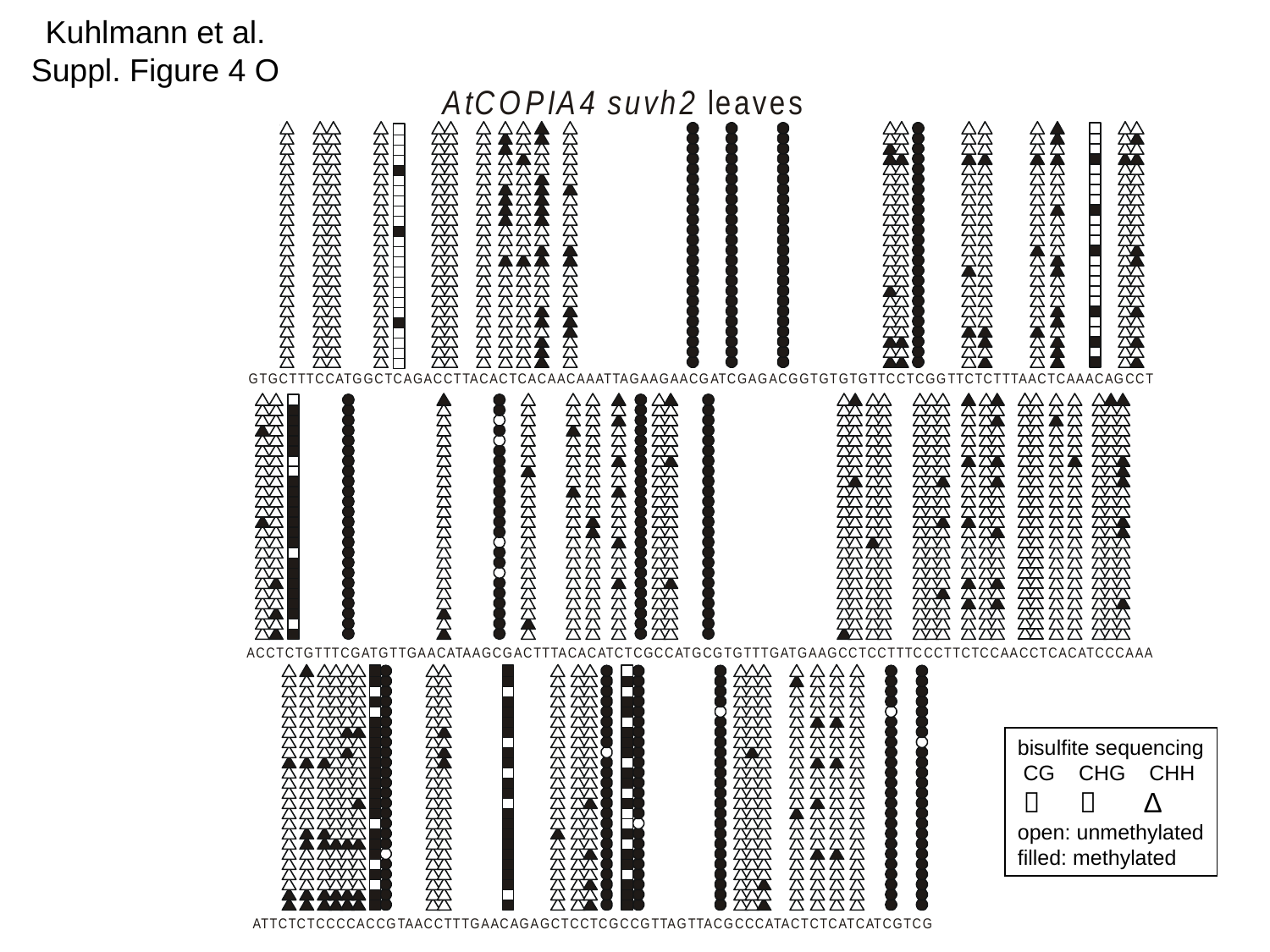

# Kuhlmann et al. Suppl. Figure 4 O
bisulfite sequencing
 CG CHG CHH
   Δ
open: unmethylated
filled: methylated

## Slide 16
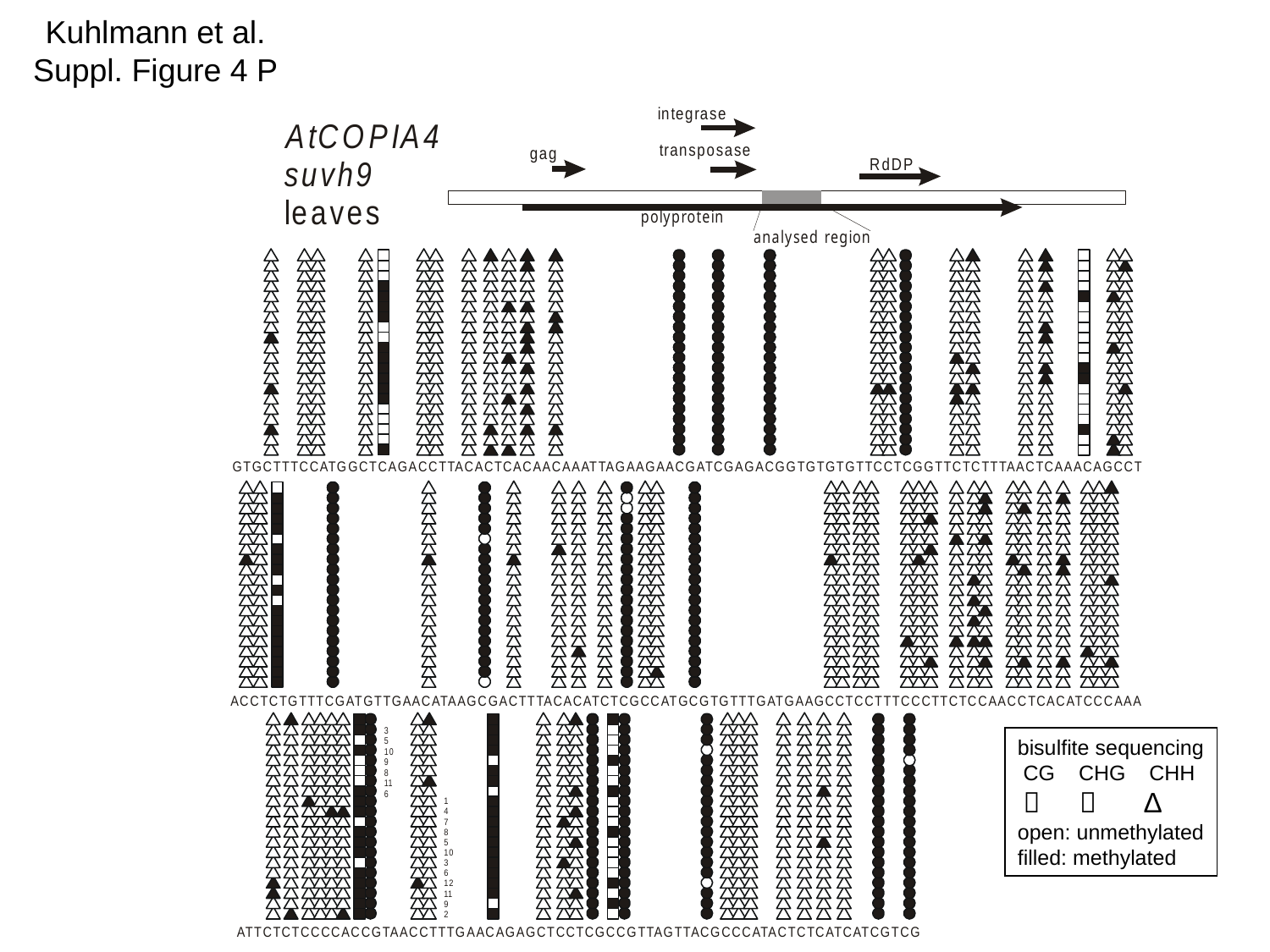

# Kuhlmann et al. Suppl. Figure 4 P
bisulfite sequencing
 CG CHG CHH
   Δ
open: unmethylated
filled: methylated

## Slide 17
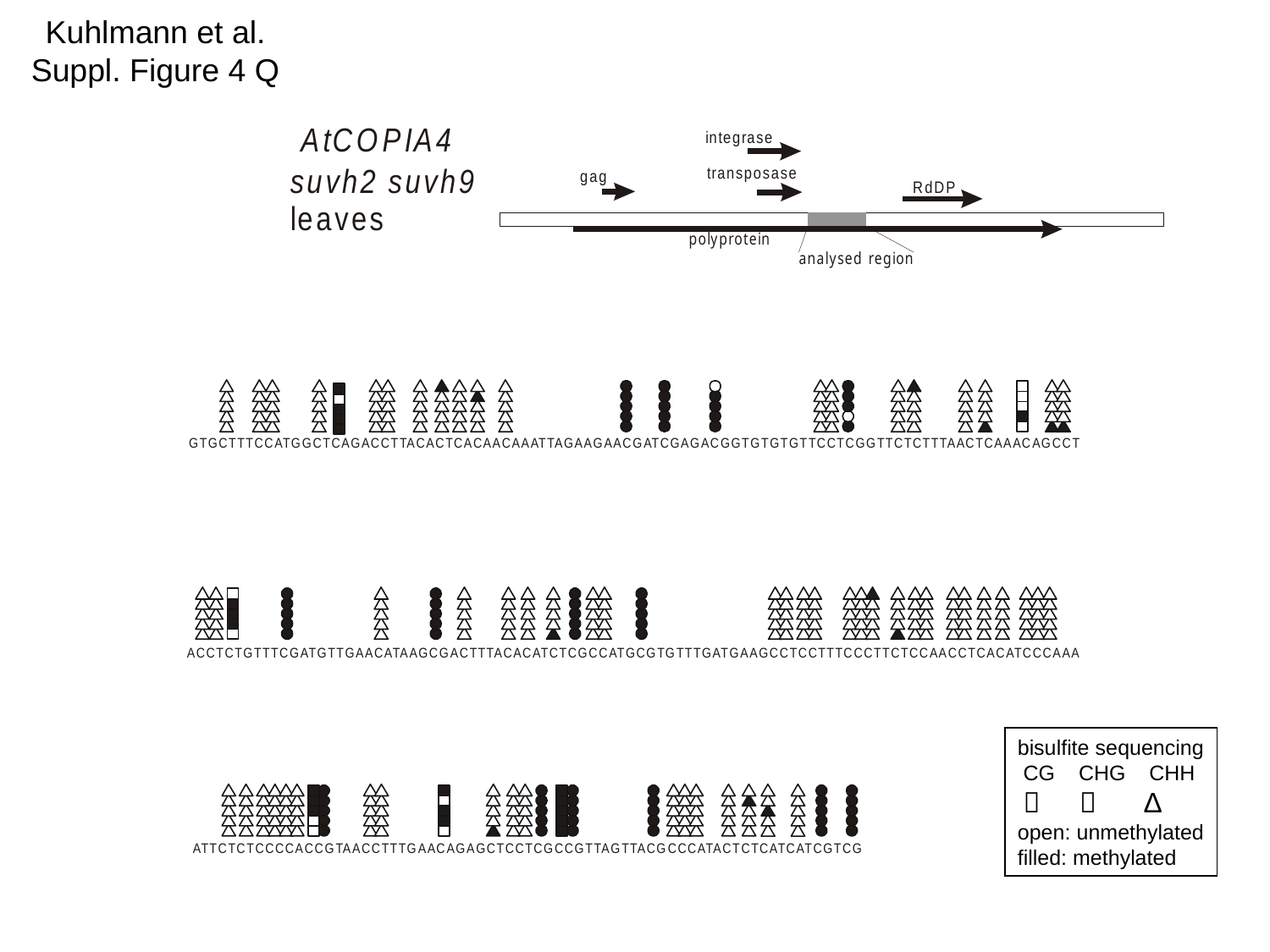

# Kuhlmann et al. Suppl. Figure 4 Q
bisulfite sequencing
 CG CHG CHH
   Δ
open: unmethylated
filled: methylated

## Slide 18
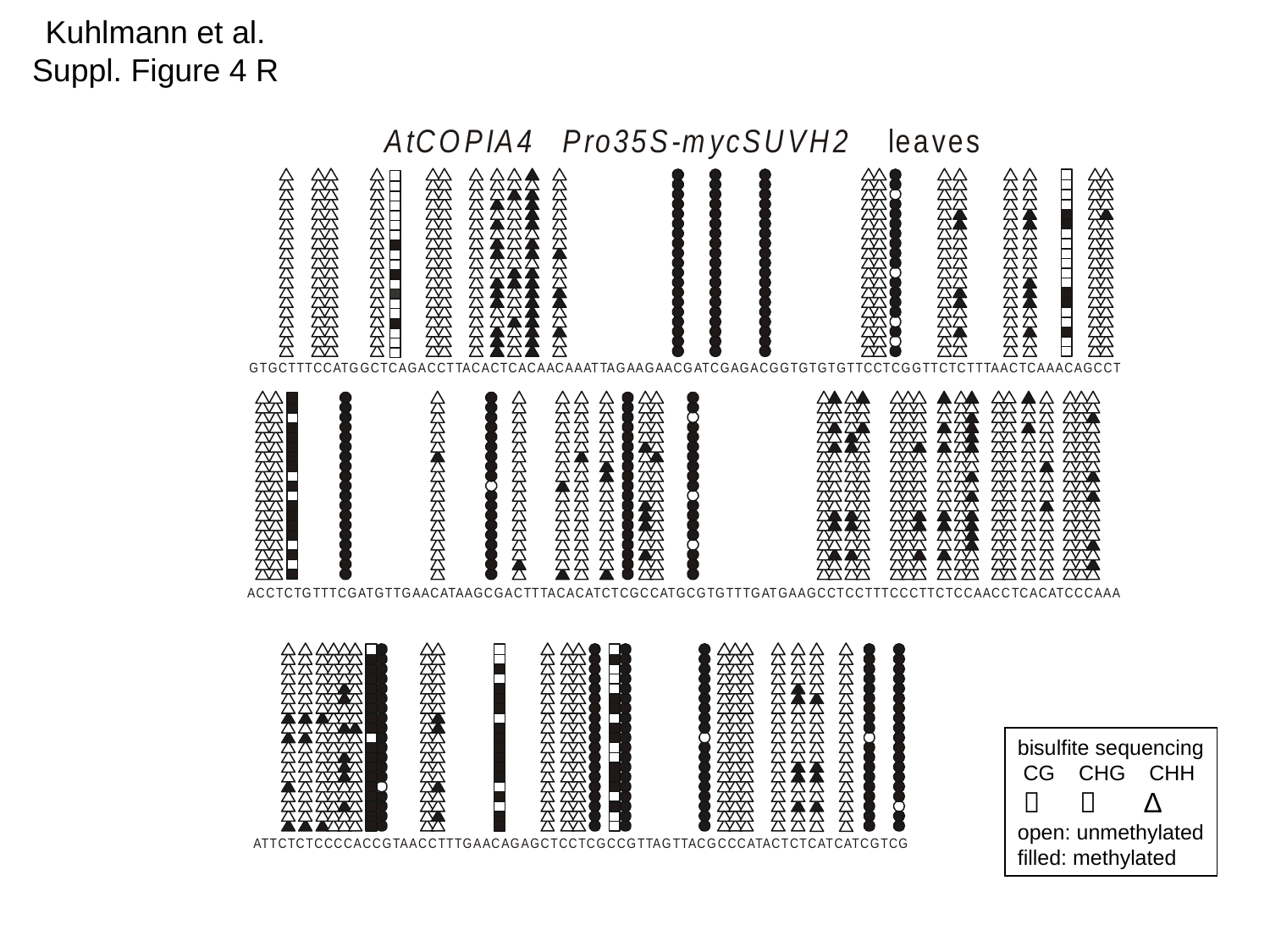

# Kuhlmann et al. Suppl. Figure 4 R
bisulfite sequencing
 CG CHG CHH
   Δ
open: unmethylated
filled: methylated
