# Supplementary material for: Developmentally non-redundant SET domain proteins SUVH2 and SUVH9 are required for transcriptional gene silencing in Arabidopsis thaliana
Source: Plant Mol Biol. 2012 Jun 6;79(6):623–33. doi: 10.1007/s11103-012-9934-x (PMC3402665; doi:10.1007/s11103-012-9934-x)
Supplement: Supplementary file 3 — Supplementary material 3 (DOCX 20 kb) [file 11103_2012_9934_MOESM3_ESM.docx]

**Supplementary Figures**

**Suppl. Figure 1**: Schematic *SUVH2* and *SUVH9* expression patterns

Expression pattern of SUVH2 (left) and SUVH9 (right) obtained from genevestigator microarray database (Hruz *et al*., 2008). The pictograms indicate the stage of development analyzed. From left: germinated seeds, seedlings, young rosette, developed rosette, bolting, young flower, developed flower, flower and silique, mature silique.

**Suppl. Figure 2**: Characterization of *suvh2* and *suvh9* T-DNA insertion lines

Scheme showing the position of available and characterized T-DNA insertions in *SUVH2* and *SUVH9*.

Yellow box indicate the SRA/YDG domain involved in DNA binding, red box indicate the SET domain. Lines with bp indicate the region amplified for quantification using quantitative real time RT PCR. Used in this study: *suvh2*: Gabi-kat_516A07 containing 2 copies of T-DNA pAC161 as inverse oriented duplication (arrows), integrated 1937bp after start ATG of At2g33290 (grey). Second allele available: SALK_079574 used in Ito *et al.,* 2011, Johnson *et al*., 2008, Naumann *et al*., 2005.

*suvh9:* SALK_048033 containing 1 copy of T-DNA pROK2 integrated in sense orientation (=>) 267 bp after start ATG of At4g13460. Allele also used in Johnson, *et al*., 2008.

**Suppl. Figure 3**: *SUVH2* and *SUVH9* transcript analysis in *suvh2*, *suvh9* and *suvh2/* *suvh9* mutants

Transcript analysis in leaf tissue by quantitative real-time RT-PCR. Col-0: wild type accession Columbia-0, *suvh2*: T-DNA insertion mutation of *SUVH2* (Gabi_kat516A07); *suvh9*: T-DNA insertion mutation of *SUVH9* (SALK_048033), *suvh2*/*suvh9*: double mutant obtained by crosses of the single mutations. The bars indicate the median of 5 independent experiments performed in triplicate relative to *PFK* mRNA (seedlings) or *ACT2* mRNA (imbibed seeds) or and the error bar the total deviation. Black bar: Col-0, yellow bar: *suvh2* (Gabi_kat516A07); orange bar: suvh9 (SALK_048033), red bar: *suvh2*/*suvh9* double mutant.

1. cDNA analysis of mutant lines for *SUVH2* mRNA relative to *PFK* mRNA.
2. cDNA analysis of mutant lines for *SUVH9* mRNA relative to *PFK* mRNA.
3. cDNA analysis of mutant lines for *ACT2* mRNA relative to *PFK* mRNA as reference gene.
4. cDNA analysis of mutant lines for *SUVH2* mRNA (yellow bar),  *SUVH9* mRNA (orange bar) and*, ACT2* mRNA (grey bar) relative to *PFK* mRNA as reference gene in seedlings.
5. cDNA analysis of mutant lines for *SUVH2* mRNA (yellow bar), ,  *SUVH9* mRNA (orange bar) and *PFK,* (grey bar) mRNA relative to *ACT2* mRNA as reference gene in 24 h imbibed seeds.

**Suppl. Figure 4**: Original data from bisulfite sequencing

1. Original *AtSN1* methylation data for wild type seedlings
2. Original *AtSN1* methylation data for *suvh2* seedlings
3. Original *AtSN1* methylation data for *suvh9* seedlings
4. Original *AtSN1* methylation data for *suvh2* *suvh9* seedlings
5. Original *AtSN1* methylation data for wild type leaves
6. Original *AtSN1* methylation data for *suvh2* leaves
7. Original *AtSN1* methylation data for *suvh9* leaves
8. Original *AtSN1* methylation data for *suvh2* *suvh9* leaves
9. Original *AtSN1* methylation data for *p35S::myc::SUVH2* leaves

J. Original *AtCOPIA4* methylation data for wild type seedlings

K. Original *AtCOPIA4* methylation data for *suvh2* seedlings

L. Original *AtCOPIA4* methylation data for *suvh9* seedlings

M. Original *AtCOPIA4* methylation data for *suvh2* *suvh9* seedlings

N. Original *AtCOPIA4* methylation data for wild type leaves

O. Original *AtCOPIA4* methylation data for *suvh2* leaves

P. Original *AtCOPIA4* methylation data for *suvh9* leaves

Q. Original *AtCOPIA4* methylation data for *suvh2* *suvh9* leaves

R. Original *AtCOPIA4* methylation data for *p35S::myc::SUVH2* leaves

**Suppl. Figure 5**: *AtSN1* and *AtCOPIA4* DNA methylation in *Pro35S-mycSUVH2* transgenic plants

DNA methylation was determined by bisulfite sequencing of genomic DNA from leaves of mature 6 week old plants. The bars mark the levels of DNA methylation in percent of methylated cytosines relative to total cytosines, with black indicating all cytosines, dark grey CG context, light grey CHG context and red indicating CHH context. Wt indicates wild type plants. **A.** DNA methylation *AtCOPIA4.* **B.** DNA methylation at in *AtSN1*.

For wild type and *35S::myc-SUVH2* over expressing plants DNA extracted from leaf tissue of 6 week old plants was used for bisulfite sequencing. The bars indicate the level of DNA methylation in %. Black bars: % of methylated cytosines relative to total cytosines in the analyzed region; dark grey bars: % methylated cytosines in CG context; light grey: % methylated cytosines in CHG context; red bars: % methylated cytosines in CHH context.

A. Analysis of DNA methylation in the *AtCOPIA4* region (Col-0: N=17; *mycSUVH2*: N=19; no sig. difference by Chi square test).

B. Analysis of DNA methylation in the *AtSN1* region (Col-0 leaf: N=22, Col-0 seedlings: N=12, *mycSUVH2*=19; no sig. difference by Chi square test).

**Suppl. Figure 6**: C: Northern Blot analysis of *AtSN1* derived siRNA.

The blot was probed with an *AtSN1* transcript A specific probe. Upper band show cross reaction of the probe with tRNA indicating homogeneity of the samples. Position of 24 mer according to NEB microRNA marker is indicated. Staining with Ethidiumbromide (EtBr) is indicating equal loading of the samples.

**Suppl. Figure 7**: Analysis of SUVH2 in RdDM by transgenic target/silencer system

1. Crossing scheme for introgression of *proNOS*::*NPTII* target gene (*K_chr1-10_,* Fischer *et al., 2008*) and *pro35S*::*proNOS* inverted repeat silencer (*H*) in homozygous *suvh2* (Gabi-kat 516A07) mutant. Plants of the F_2_ generation were selected for further analysis based on genotyping by PCR.
2. *NPTII* mRNA expression analysis relative to *ACT2* mRNA determined by RT qPCR. Bars indicate the median of 5 independent analyzed plants and the given error bars indicate the total deviation. *K*- target transgene, *H*-silencer transgene, *suvh2*-*suvh2*-allel (Gabi-kat 516A07)
3. DNA methylation analysis of *proNOS* by Southern blot analysis with methylation sensitive restriction enzymes and quantification of DNA methylation by bisulfite sequencing. Upper part: Southern analysis performed according Fischer *et al*., (2008). Lower part: Quantification of DNA methylation by bisulfite sequencing of the *proNOS*. Black bar: percent of methylated cytosine relative to total cytosines in the analyzed region; dark grey bar: percent of methylated cytosine in a CG context; bright grey bar: percent of methylated cytosine in a CHG context; red bar: percent of methylated cytosine in a CHH context. (Number of sequences analyzed: K/-, -/-, SUVH2/SUVH2: N=7 ; K/-, H/-, SUVH2/SUVH2: N=7 ; K/-, -/-, *suvh2*/*suvh2*: N=18 ; K/-, H/-, *suvh2*/*suvh2*: N=16).
4. Northern blot analysis of *proNOS* siRNA (left) and dsRNA (right)

Blots were probed with specific *proNOS* RNA probes according to Mette *et al*. (2005) and Fischer *et al*. (2008).

**Suppl. Figure 8**: PCR efficiencies in qPCR analysis.

Depicted are the PCR efficiency and Correlation Coefficient for qPCRs used for amplification of *SUVH2*, *SUVH9*, *ACT2* and *PFK* mRNA.

**Suppl. Table**: Sequences of oligonucleotides used in this study
